# Supplementary material for: HaloTag-based approach to quantify subcellular localization of TRPV3 channels
Source: Biophys J. 2026 Mar 4;125(7):1667–85. doi: 10.1016/j.bpj.2026.03.003 (PMC13351909; doi:10.1016/j.bpj.2026.03.003)
Supplement: Document S1. Figures S1–S7 [file mmc1.pdf]

Biophysical Journal, Volume *125*

Supplemental information

HaloTag-based approach to quantify subcellular localization of TRPV3 channels

Alexander Holloway, Joshua Chiang, Afroza Khan, Eric N. Senning, and Andrés Jara-Oseguera

SUPPORTING MATERIAL

HaloTag-based approach to quantify subcellular localization of TRPV3 channels.

Alexander Holloway, Joshua Chiang, Afroza Khan, Eric N. Senning, and Andrés Jara-Oseguera.

<u>Contents</u>:

Figure S1

Figure S2

Figure S3

Figure S4

Figure S5

Figure S6

Figure S7

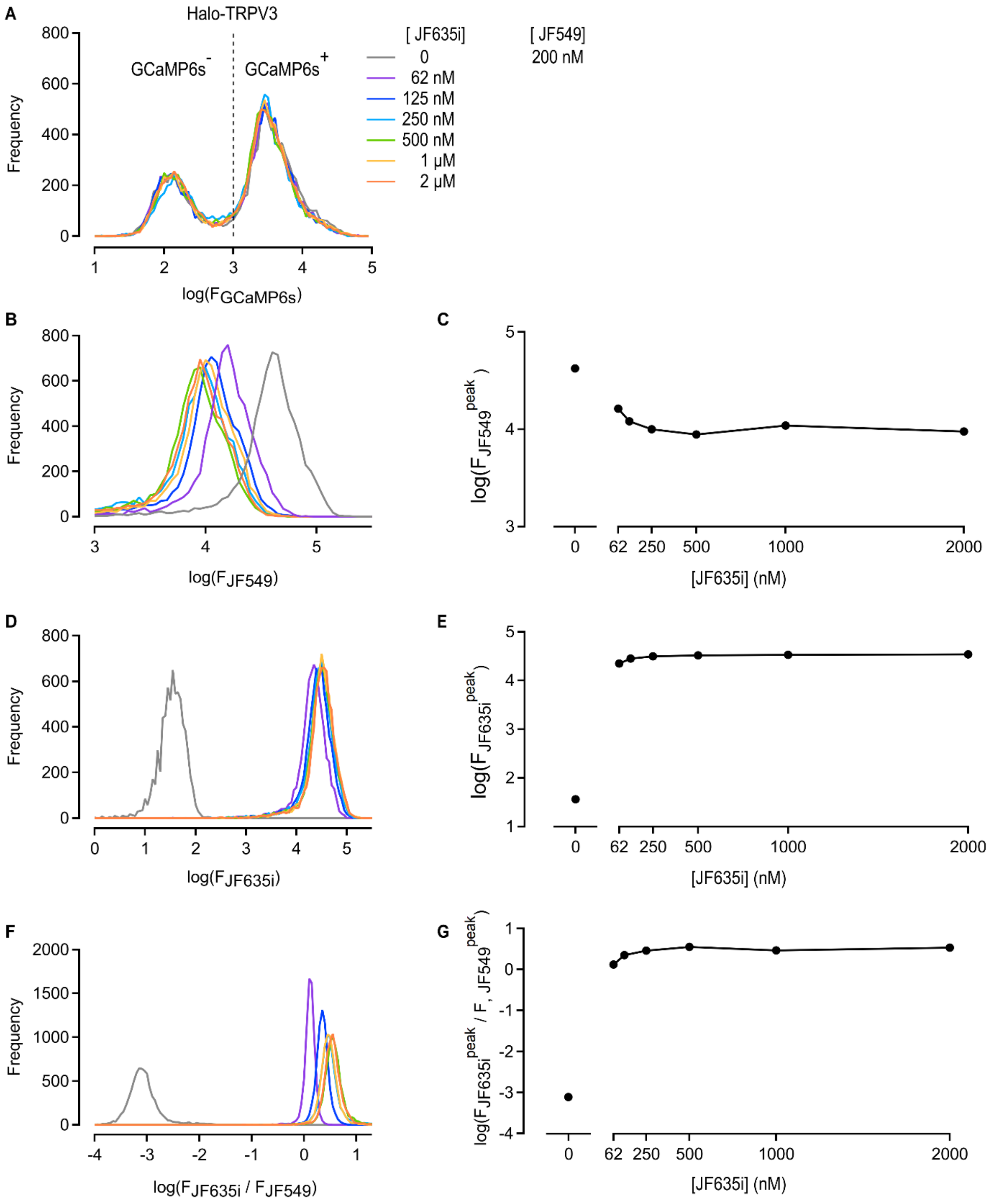

Figure S1. Labeling titration of surface-exposed Halo-TRPV3 channels with JF635i. Quantitative comparison of seven populations of cells expressing Halo-TRPV3 channels titrated

with JF635i at a concentration of 0 nM, 62 nM, 125 nM, 250 nM, 500 nM, 1 μM, or 2 μM before subsequent labeling with JF549 at a concentration of 200 nM. Cells were analyzed by flow cytometry and the Gaussian means of the corresponding histograms were plotted on a saturation curve. **(A, B, D)** Histograms showing the fluorescence intensity distributions for GCaMP6s (A), JF549 (B), and JF635i (D) of cells expressing Halo-TRPV3, measured by flow cytometry. Cells were labeled with 200 nM JF549 and different concentrations of JF635i, as indicated by the trace colors. A total of 1 x 10^4 cells were analyzed per experimental condition. Histograms in A contain data for all cells, and histograms in (B) and (D) display only data for $GCaMP6s^+$ cells. The dashed line in (A) indicates the GCaMP6s fluorescence intensity cutoff to select for $GCaMP6s^+$ cells in (B) and (D). **(C, E)** JF635i label titration curves of JF549 (C) and JF635i (E) peak fluorescence intensity as a function of the concentration of JF635i used for labeling, obtained from fits of a Gaussian function to the histograms in (B) or (D). **(F)** Histograms of the logarithmic ratio between JF549 and JF635i fluorescence intensities, calculated from the data in (B) and (D). **(G)** JF635i label titration curves of the peak logarithmic ratio between the fluorescence intensities of JF549 and JF635i as a function of the JF635i dye concentration, obtained from fits of a Gaussian function to the histograms in (F).

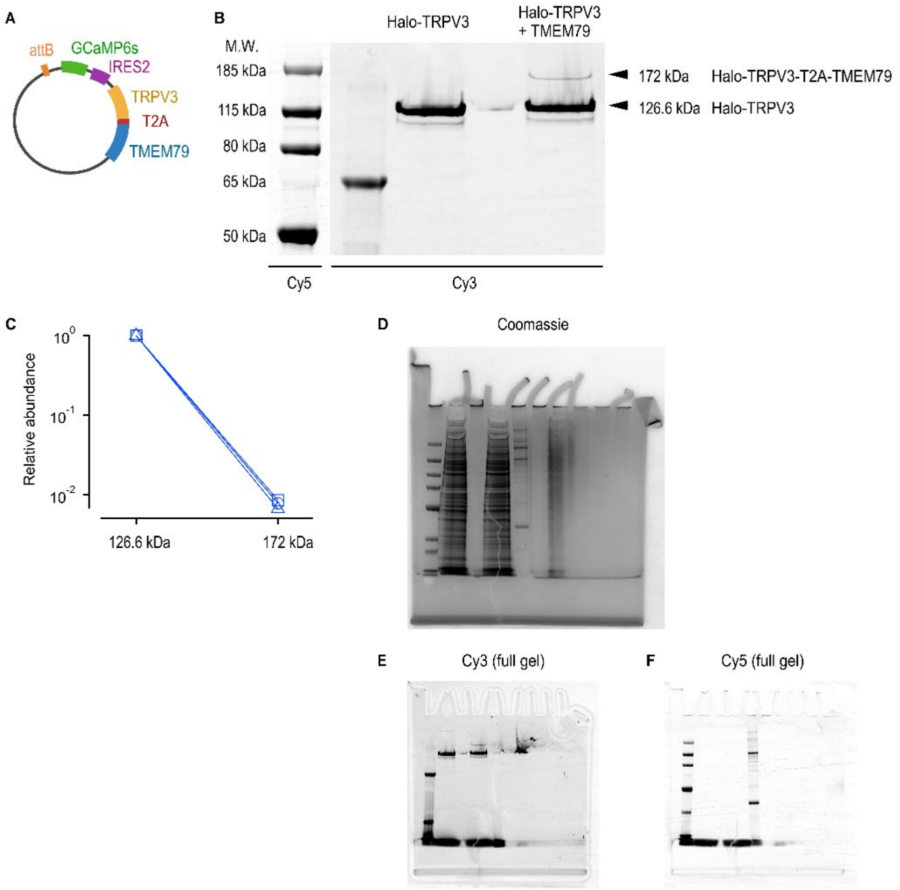

Figure S2. Validation of the use of a T2A sequence to co-express Halo-TRPV3 and TMEM79 in landing pad cells. (A) Cartoon of the promoter-less plasmid that we used to express GCaMP6s, Halo-TRPV3, and TMEM79 in iCasp9 landing pad cells (52). A T2A sequence (dark red) was introduced between the TRPV3 and TMEM79 coding sequences. **(B)** Representative in-gel fluorescence of the total cell lysate obtained from cells expressing Halo-TRPV3 or Halo-TRPV3-T2A-TMEM79 and that were labeled with JF549. The arrows denote bands with molecular weights (M.W.) corresponding to a single Halo-TRPV3 subunit or a Halo-TRPV3-T2A-TMEM79 fusion protein. The molecular weight marker on the same gel was imaged in the Cy5 channel (left panel) and the Cy3 channel (first lane on the right panel). **(C)** Relative abundance of the single

Halo-TRPV3 protein and the Halo-TRPV3-T2A-TMEM79 fusion protein relative to the total labeled Halo-TRPV3 in the gel for lysates of cells recombined with the plasmid for TMEM79 co-expression. Data for three replicate experiments are shown. **(D)** Coomassie staining of the same gel as in (B). **(E)** In-gel fluorescence for the whole gel shown in (B) imaged on the Cy3 channel that shows JF549 fluorescence. **(F)** In-gel fluorescence for the whole gel shown in (B) imaged on the Cy5 channel that shows most bands of the M.W. marker.

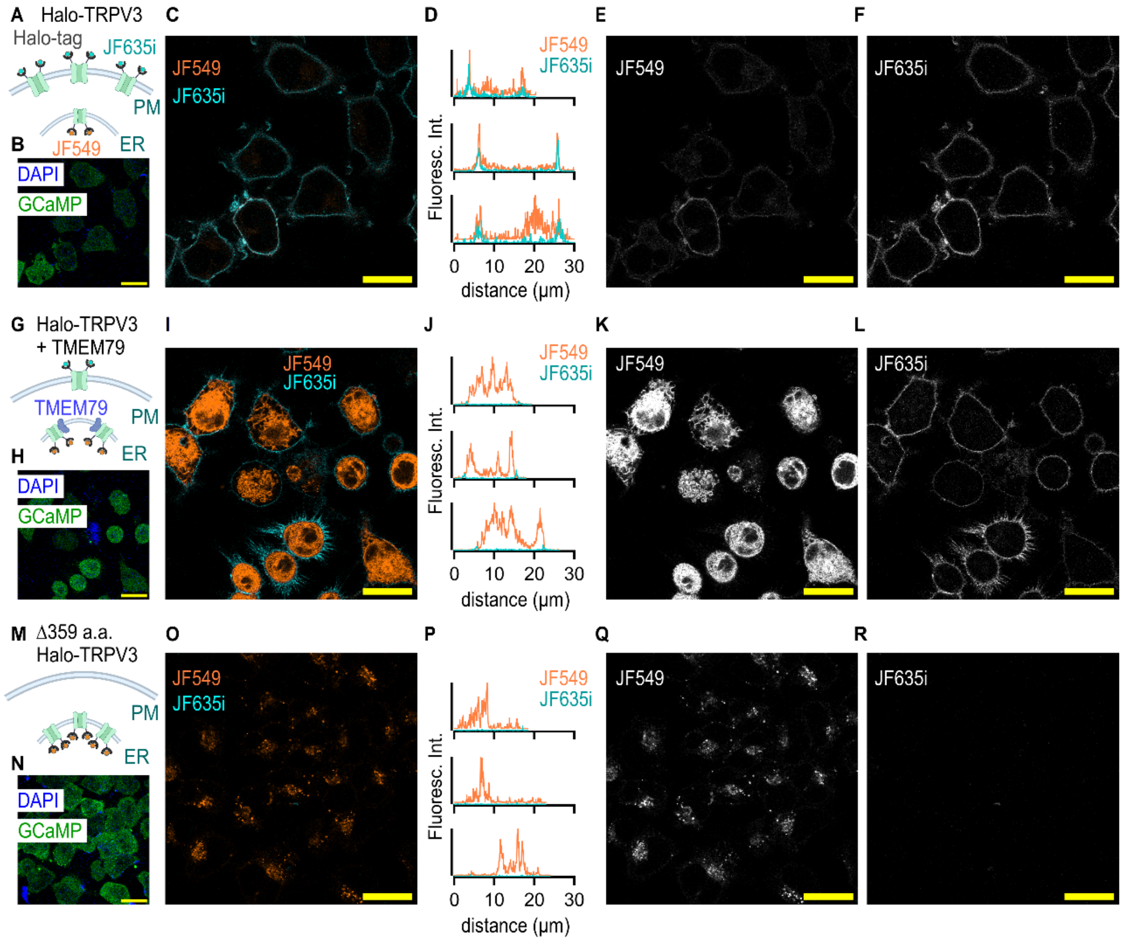

Figure S3. Visualization of Halo-TRPV3 subcellular localization by confocal microscopy in fixed cells. (A, G, and M) Cartoons depicting subcellular localization of Halo-TRPV3 (A), Halo-TRPV3 + TMEM79 (G), and Halo-TRPV3 Δ359 a.a. (M) as determined by our labeling approach applied to fixed cells, using a membrane impermeable dye (cyan, JF635i) followed by a membrane-permeable dye (orange, JF549). PM stands for plasma membrane and ER for endoplasmic reticulum and other intracellular compartments. **(B, H, and N)** Representative GCaMP6s (green) and DAPI (blue) overlaid confocal microscopy images of cells that were fixed before labeling with JF635i, JF549, and DAPI. Images were acquired using a 60x objective. Scale

bars = 20 μm. **(C, I, and O)** Representative JF549 (orange) and JF635i (cyan) overlaid confocal microscopy images of the same fields of view as in (B, H, and N). **(D, J, and P)** JF635i (cyan) and JF549 (orange) fluorescence intensity line profiles from three representative confocal microscopy images of fixed cells expressing Halo-TRPV3 (D), Halo-TRPV3 + TMEM79 (J), or Halo-TRPV3 Δ359 a.a. (P). **(E, K, and Q)** Representative JF549 confocal microscopy images of the same fields of view as in (B, H, and N). **(F, L, and R)** Representative JF635i confocal microscopy images of the same fields of view as in (B, H, and N).

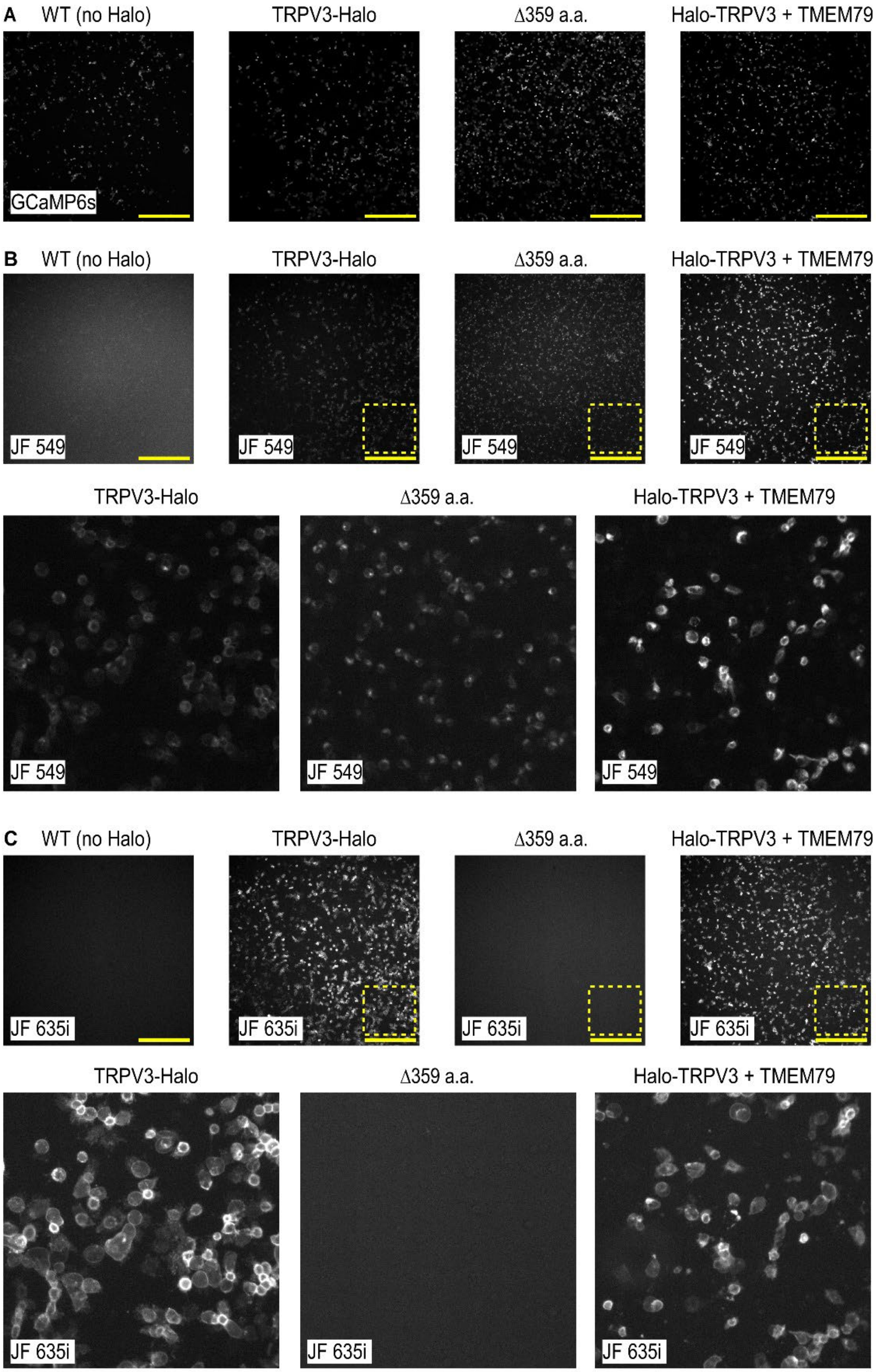

Figure S4. Visualization of Halo-TRPV3 subcellular localization by epifluorescence microscopy in live cells. (A) Representative GCaMP6s fluorescence images obtained by

epifluorescence microscopy of live cells labeled with JF635i and JF549. Images were acquired using a 10x objective. Scale bar is 400 µm. **(B)** Representative JF549 fluorescence images obtained by epifluorescence microscopy of live cells labeled with JF635i and JF549. Images were acquired using a 10x objective. The bottom panel shows magnified images of the regions denoted by the dashed yellow boxes. **(C)** Representative JF635i fluorescence images obtained by epifluorescence microscopy of live cells labeled with JF635i and JF549. Images were acquired using a 10x objective. The bottom panel shows magnified images of the regions denoted by the dashed yellow boxes.

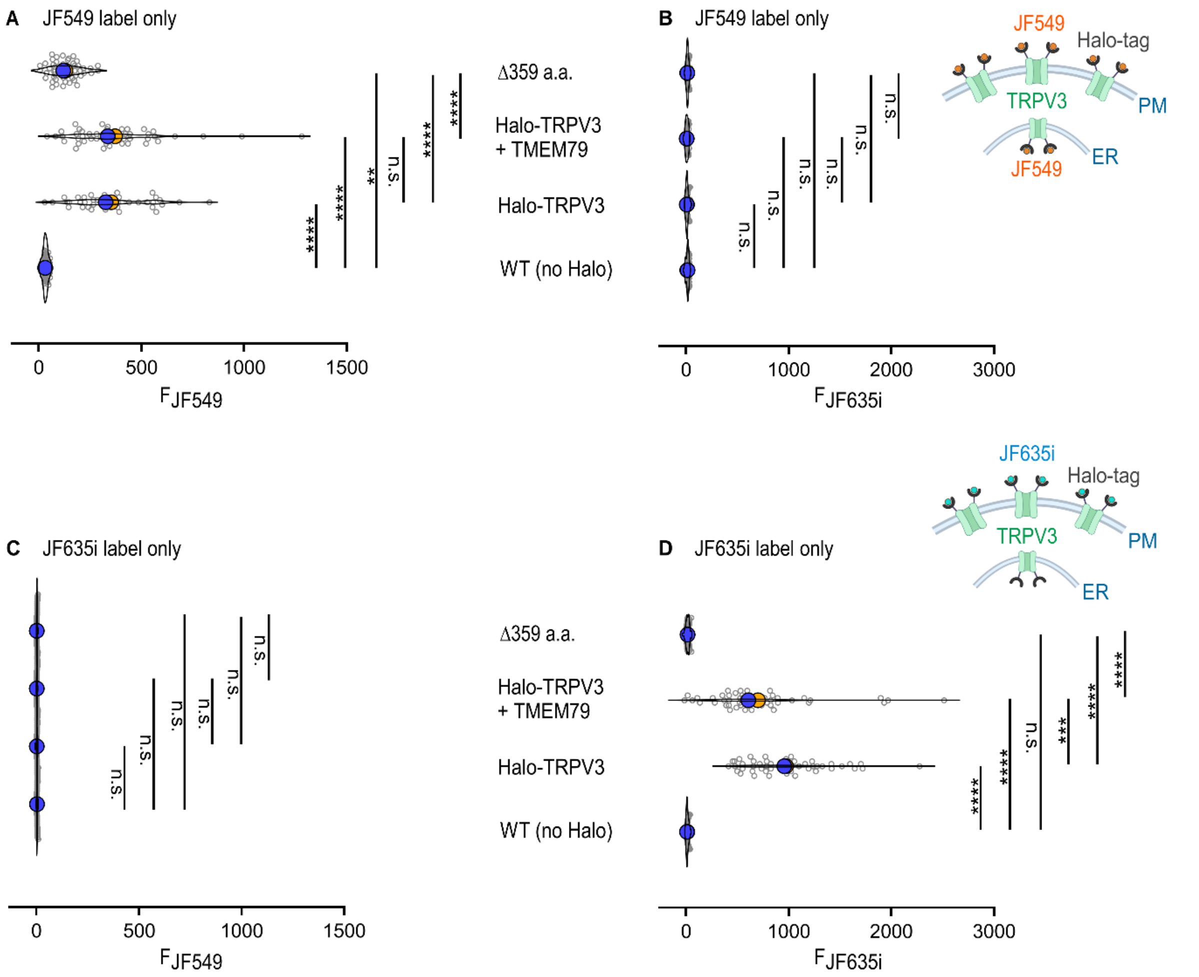

Figure S5. Quantitation of surface-expressed vs intracellular Halo-TRPV3 channels by epifluorescence microscopy in cells labeled with just one of the two dyes. (A) Violin plots of the mean JF549 fluorescence intensity of individual cells measured from epifluorescence microscopy images of cells labeled with JF549 only. Plots contain data from a single experiment, where fifty cells were randomly chosen based on the presence of GCaMP6s fluorescence. Data for individual cells are shown as open gray circles, and the mean and median are shown as yellow and blue circles, respectively. Statistical significance was evaluated using a Tukey HSD test (Halo vs WT, $p = 2.20 \times 10^{-13}$; Halo vs Δ359, $p = 3.84 \times 10^{-10}$; Halo vs TMEM79, $p = 0.93$; TMEM79 vs WT, $p = 1.77 \times 10^{-14}$; TMEM79 vs Δ359, $p = 2.71 \times 10^{-11}$; WT vs Δ359, $p = 0.0054$). **(B)** Violin

plots of the mean JF635i fluorescence intensity of the same cells as in (A). Data are depicted as in (A). Statistical significance was evaluated using a Tukey HSD test (Halo vs WT, $p = 0.917$; Halo vs Δ359, $p = 0.68$; Halo vs TMEM79, $p = 0.645$; TMEM79 vs WT, $p = 0.22$; TMEM79 vs Δ359, $p = 0.075$; WT vs Δ359, $p = 0.959$). **(C)** Violin plots of the mean JF549 fluorescence intensity of individual cells measured from epifluorescence microscopy images of cells labeled with JF635i only. Plots contain data from a single experiment, where fifty cells were randomly chosen based on the presence of GCaMP6s fluorescence. Data are depicted as in (A). Statistical significance was evaluated using a Tukey HSD test (Halo vs WT, $p = 0.533$; Halo vs Δ359, $p = 0.977$; Halo vs TMEM79, $p = 0.752$; TMEM79 vs WT, $p = 0.092$; TMEM79 vs Δ359, $p = 0.934$; WT vs Δ359, $p = 0.299$). **(B)** Violin plots of the mean JF636i fluorescence intensity of the same cells as in (C). Data are depicted as in (A). Statistical significance was evaluated using a Tukey HSD test (Halo vs WT, $p = 7.57 \times 10^{-14}$; Halo vs Δ359, $p = 7.57 \times 10^{-14}$; Halo vs TMEM79, $p = 0.000169$; TMEM79 vs WT, $p = 1.72 \times 10^{-13}$; TMEM79 vs Δ359, $p = 1.86 \times 10^{-13}$; WT vs Δ359, $p = 1$).

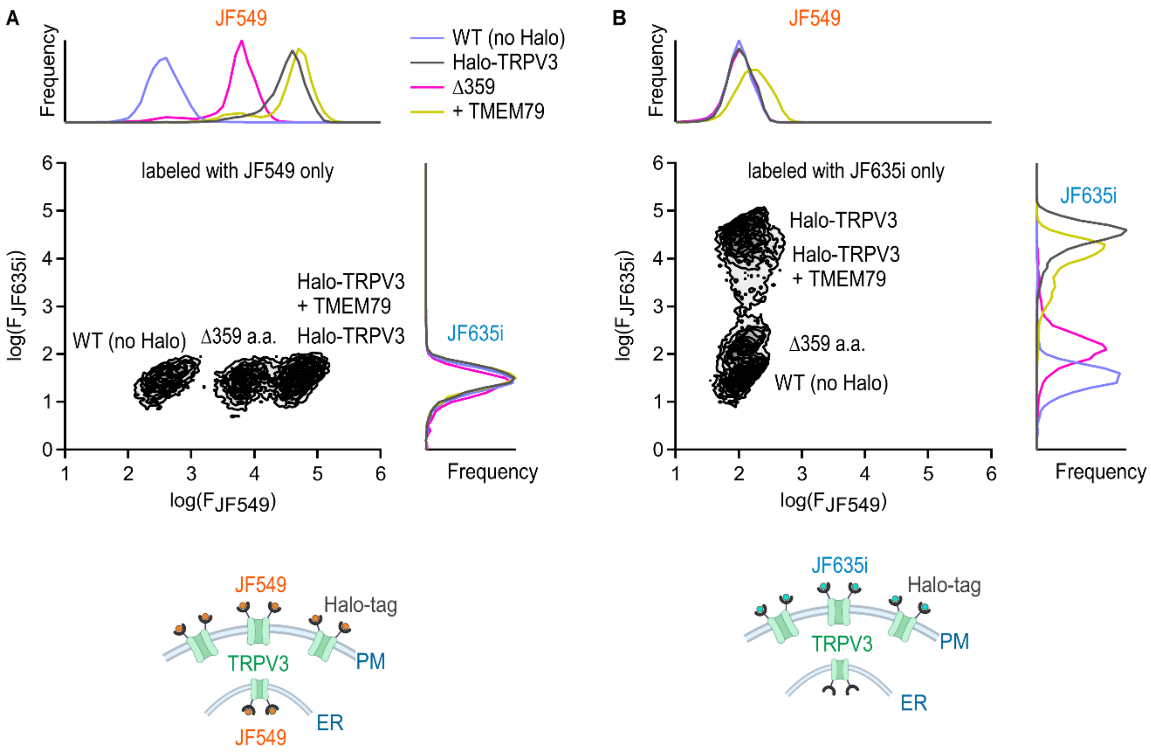

Figure S6. Quantitation of surface-expressed vs intracellular Halo-TRPV3 channels by flow cytometry in cells labeled with just one of the two dyes. (A, top panel**)** Histograms showing the logarithmic JF549 fluorescence intensity distributions of single GCaMP6s$^+$ cells labeled with JF549 only and measured by flow cytometry. Data for a single experiment is shown, with 1×10^4 analyzed GCaMP6s$^+$ single cells. **(A**, right panel**)** Histograms showing the logarithmic JF635i fluorescence intensity distributions measured by flow cytometry of the same cells as in the top panel. **(A**, center panel**)** Surface plots depicting the cell distribution as a function of their JF549 and JF635i fluorescence intensities. **(B**, top panel**)** Histograms showing the logarithmic JF549 fluorescence intensity distributions of single GCaMP6s$^+$ cells labeled with JF635i only and measured by flow cytometry. Data for a single experiment is shown, with 1×10^4 analyzed GCaMP6s$^+$ single cells. **(B**, right panel**)** Histograms showing the logarithmic JF635i fluorescence intensity distributions measured by flow cytometry of the same cells as in the top panel. **(B**, center

panel**)** Surface plots depicting the cell distribution as a function of their JF549 and JF635i fluorescence intensities.

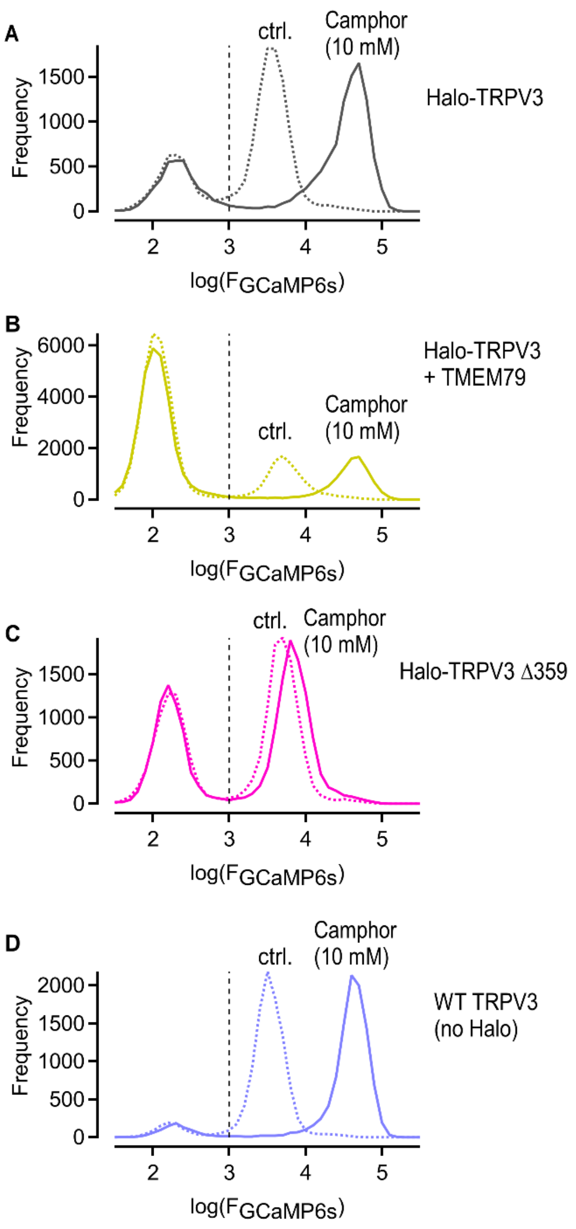

Figure S7. Measuring the response of TRPV3 channels to camphor with a fluorescent calcium reporter. (A-D) Histograms showing the GCaMP6s fluorescence intensity distribution in the presence (solid curves) or absence (dotted curves) of 10 mM camphor of unlabeled cells expressing Halo-TRPV3 (A, gray), Halo-TRPV3 + TMEM79 (B, lime green), Halo-TRPV3 Δ359 a.a. (C, pink), and WT TRPV3 (no Halo) (D, light blue). A total of 1×10^4 cells were analyzed per

experimental group. The dashed vertical line distinguishes between GCaMP6s^{-} and GCaMP6s^{+} cell populations.
