# Supplementary material for: HaloTag-based approach to quantify subcellular localization of TRPV3 channels
Source: Biophys J. 2026 Mar 4;125(7):1667–85. doi: 10.1016/j.bpj.2026.03.003 (PMC13351909; doi:10.1016/j.bpj.2026.03.003)
Supplement: Document S2. Article plus supporting material [file mmc2.pdf]

Biophysical Journal
Article

Biophysical Society

HaloTag-based approach to quantify subcellular localization of TRPV3 channels

Alexander Holloway,[1] Joshua Chiang,[1] Afroza Khan,[1] Eric N. Senning,[2] and Andrés Jara-Oseguera[1,*]
[1]Department of Molecular Biosciences, College of Natural Sciences, The University of Texas at Austin, Austin, Texas and [2]Department of Neuroscience, College of Natural Sciences, The University of Texas at Austin, Austin Texas

ABSTRACT The TRPV3 channel is crucial for skin barrier formation and hair growth, and its dysregulation is linked to itch, atopic dermatitis, rosacea, and genetic disorders like Olmsted syndrome. Heat and voltage directly activate TRPV3 channels to conduct cations through their pore. In addition, interactions between TRPV3 and the transmembrane protein TMEM79 influence channel trafficking rather than pore gating. Otherwise, little is known about how the TRPV3 channel is endogenously controlled. Importantly, we lack the experimental tools to reliably quantify TRPV3 channel cell surface expression under diverse experimental conditions, including live cells. To address this gap in knowledge, we successfully fused a cpHaloTag to the extracellular face of the mouse TRPV3 channel while retaining its sensitivity to voltage, heat, and agonists. We show that we can differentially detect surface-expressed and intracellularly localized channels in HEK293 cells by sequentially labeling with spectrally separable membrane-permeable and impermeable HaloTag dyes. Using this tool and confocal microscopy, we detect robust changes in TRPV3 channel localization on the cell surface of live and fixed cells caused by truncation of an N-terminal portion of the channel or by its co-expression with TMEM79. Notably, we show that these changes in TRPV3 channel subcellular localization can be robustly quantified by epifluorescence microscopy at low magnification and flow cytometry, making our tool ideal for high-throughput screening applications. Furthermore, we co-express Halo-TRPV3 channels together with a fluorescent Ca^{2+}-reporter, plasma membrane-, or lysosomal-fluorescent markers and show that this system can be used to assess channel activity or organellar localization. This work thus establishes the practicality and sensitivity of a flexible new tool to elucidate the molecular and cellular mechanisms of TRPV3 channel function.

SIGNIFICANCE We describe a HaloTag-TRPV3 channel fusion protein that makes it possible to separate the contribution of cell surface expression and channel gating to overall TRPV3 channel activity. This tool can aid future ion channel studies by enabling the screening of cell libraries expressing channel mutants or containing gene knockouts to identify perturbations that affect channel subcellular localization or gating. As a knockin in transgenic animals or human stem cells, the flexibility and enhanced signal of HaloTag in this tool would be transformative for learning how this channel is regulated in vivo, especially in cell types like neurons or cardiac cells, where low expression of TRPV3 has made it challenging to detect channel expression or activity reliably.

INTRODUCTION

TRPV3 is a nonselective cation-permeable ion channel that belongs to the vanilloid subfamily of transient receptor potential (TRP) channels. It assembles as a homotetramer, with each subunit comprising six transmembrane helices (S1–S6) and large cytoplasmic N- and C-terminal domains with six N-terminal ankyrin repeats (1–5). Expressed primarily in skin keratinocytes (6), TRPV3 channels play a role in skin thermosensation (7–10), pain and inflammation (11–14), itch (15–17), as well as skin barrier formation and homeostasis (18), wound healing (19,20), and hair growth (18,21). The TRPV3 is reported to be expressed in sensory neurons (1,22), the brain (23–25), and the heart (26,27), but its function there is less clear because its low expression in those cells and tissues makes it difficult to study. TRPV3 channels can be directly activated by voltage and heat (1,6), plant monoterpenes (28,29), cannabinoids (30), and synthetic organic molecules like 2-aminoethoxydiphenyl borate (2-APB) (31,32) through molecular mechanisms that are not yet understood. In addition, TRPV3 channels

Submitted September 24, 2025, and accepted for publication March 2, 2026.

*Correspondence: andres.jaraoseguera@austin.utexas.edu

Alexander Holloway and Joshua Chiang contributed equally to this work.

Alexander Holloway's present address is Baylor College of Medicine, Houston, Texas.

Editor: Valeria Vasquez.

https://doi.org/10.1016/j.bpj.2026.03.003

© 2026 The Author(s). Published by Elsevier Inc. on behalf of Biophysical Society.
This is an open access article under the CC BY license (http://creativecommons.org/licenses/by/4.0/).

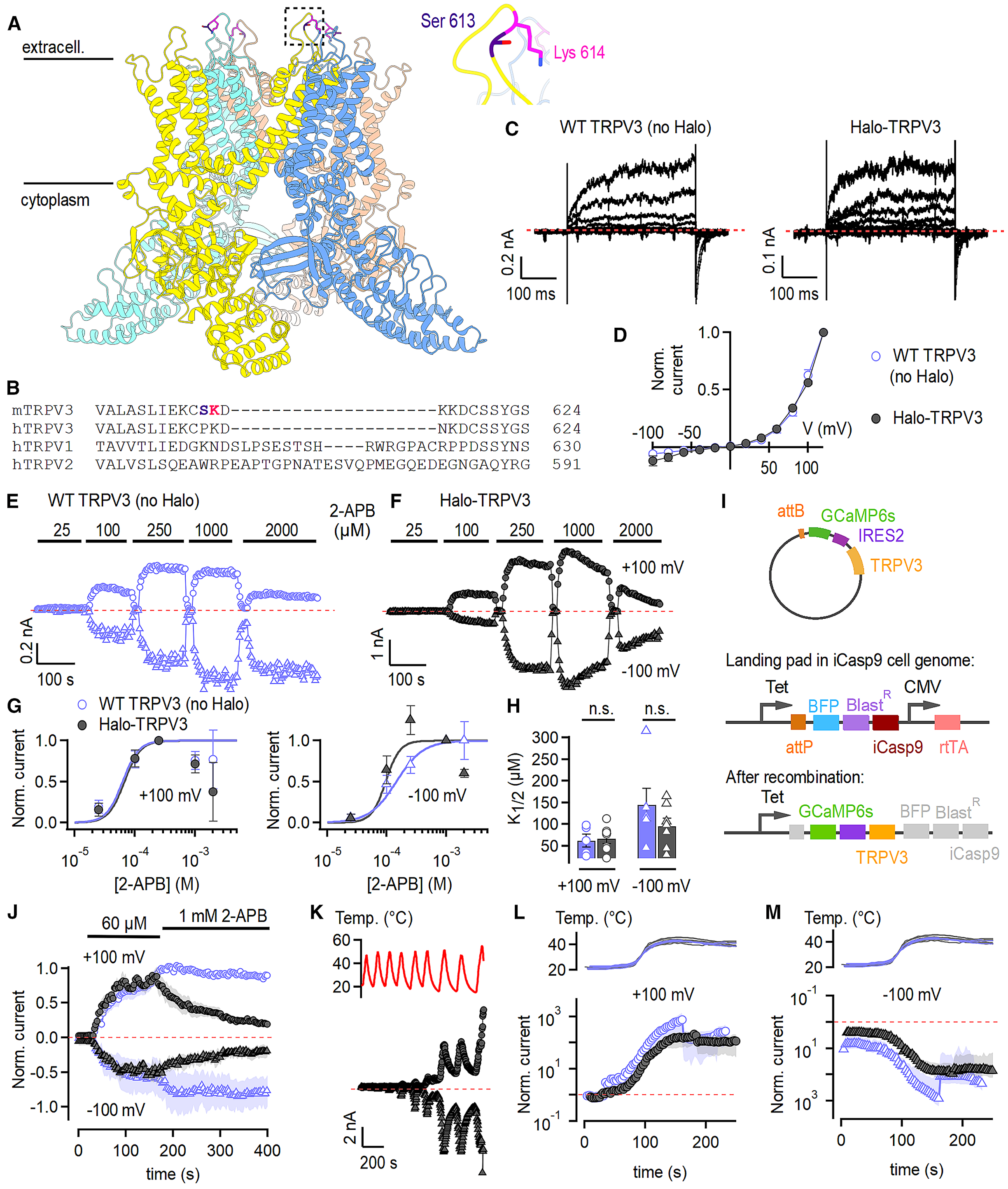

FIGURE 1 A functional HaloTag-TRPV3 fusion protein for interrogating channel subcellular localization. (*A*) Cartoon representation of an atomic model for the mouse TRPV3 channel complex (PDB: 7MIJ) (51) with a magnified view of the HaloTag insertion site (*dashed box*) between Ser 613 (*purple*) and Lys 614 (*magenta*). (*B*) Amino acid sequence alignment of the region flanking the HaloTag insertion site between Ser 613 (*purple*) and Lys 614 (*magenta*) in mTRPV3. (*C*) Representative whole-cell current families elicited at room temperature by voltage steps from −100 to +120 mV from a holding potential of −60 mV. The dashed red line denotes the zero-current level. (*D*) Normalized current-voltage relations obtained from experiments as in (*C*). Data are shown as mean ± SEM ($n = 4$–5). (*E* and *F*) Representative whole-cell current time courses elicited by voltage steps between −100 (triangles) and +100 mV (circles) while the patch-clamped cells were exposed to increasing concentrations of 2-APB, as denoted by the black vertical lines. The dashed red line denotes the zero-current level. (*G*) Concentration-response relations for 2-APB obtained from experiments as in (*E*) and (*F*) using the currents measured at +100 (*left*, circles) or −100 mV (*right*, triangles). Data shown as mean ± SEM ($n = 5$–7). Continuous curves are fits to the Hill equation. (*H*) $K_{1/2}$ values for channel activation by 2-APB obtained from the data in (*G*). Bars depict the mean ± SEM, and the open symbols are parameters from individual experiments. No statistically significant differences were

(*legend continued on next page*)

and the epidermal growth factor receptor (EGFR) form a signaling complex in the plasma membrane of skin keratinocytes that positively regulates TRPV3 channel activity via phosphorylation (18). Other mechanisms can influence channel localization rather than activity, such as the direct interaction between TRPV3 and the membrane protein TMEM79 or the Sorting Nexin 11 (SNX11) protein, both of which reduce channel expression in the plasma membrane and promote TRPV3 degradation in lysosomes (9,33). Regulation by both EGFR and TMEM79 is necessary for normal skin physiology, and the interaction of TRPV3 with SNX11 is suggested to contribute to skin thermo-sensitivity in mice (34). Notably, there is limited information about the endogenous mechanisms that control TRPV3 channel function under physiological and pathophysiological conditions. A deeper understanding of the molecular mechanisms that regulate TRPV3 function and expression would facilitate the development of therapeutic interventions to counteract disorders resulting from the dysregulation of these channels, which include severe genetic skin disorders like Olmsted syndrome (35–37).

A major limitation toward learning how TRPV3 channels are regulated is the lack of tools to detect and quantify TRPV3 channel expression in the plasma membrane of live cells or animals. Antibodies against the extracellular domains of TRP channels demonstrate low specificity. Surface biotinylation, on the other hand, requires large numbers of cells that must all have an intact plasma membrane to yield optimal results, because cells are biotinylated and processed in bulk. The sensitivity of this approach is further impacted by the multiple steps required for analysis, which include the blot transfer of proteins into a membrane, and the need of primary and secondary antibodies for protein visualization. One powerful tool for studying subcellular protein localization is the HaloTag (38), (39–41). The HaloTag is a globular protein derived from a bacterial haloalkane dehalogenase enzyme that binds substrates specifically, efficiently, and irreversibly. This tool is modular and flexible, with a wide variety of available functionalized ligands, such as fluorescent dyes with a diverse range of properties, affinity tags, or enzyme substrates (41–43). Fusion of a HaloTag to an additional engineered N-terminal transmembrane helix in voltage-gated Na^+ (Na_v) channels generated a construct compatible with extracellular labeling that has been used in combination with diverse fluorescence labeling schemes involving membrane-permeable and -impermeable HaloTag dyes to understand how Na_v channel trafficking is regulated in sensory neurons (44–48). Successful fluorescent labeling of TRPV1 channels fused to an extracellular circularly permuted (cp) HaloTag (49) between transmembrane segments has also been demonstrated (50). Notably, the circular permutation brings the N- and C-termini of the HaloTag closer together, making it less disruptive as a fusion protein when inserted within loops of adjacent transmembrane helices.

By inserting a circularly permuted (cp) HaloTag (49), which we will refer to simply as HaloTag in the rest of this manuscript, into the extracellular loop between the fifth and sixth transmembrane domains (S5–S6) of the mouse TRPV3 channel (Fig. 1, *A* and *B*), we show that it is possible to selectively label surface-expressed TRPV3 channels using a membrane-impermeable fluorescent dye. Furthermore, subsequent labeling with a spectrally separable membrane-permeable dye allows for specific labeling of intracellularly localized TRPV3. This enables the precise quantitation of relative protein abundance between the plasma membrane and intracellular compartments using live-cell imaging or flow cytometry. Furthermore, our system allows the interrogation of channel activity by co-expressing channels together with a spectrally separable fluorescent Ca^{2+}-reporter or the identification of Halo-TRPV3 channel expression in specific subcellular compartments using spectrally separable organellar markers. This tool can be used to screen libraries of TRPV3 channel mutants or utilized as a knockin in genetically modified animals or human pluripotent stem cells, providing a new approach to understanding and therapeutically influencing the molecular mechanisms underlying TRPV3 channel activity.

MATERIALS AND METHODS

Molecular cloning and construct design

The mouse TRPV3 coding sequence was purchased from Twist Bioscience as a clonal gene integrated into the promoterless KAM05_attB_mCherry vector, which was a gift from Doug Fowler (University of Washington). A fragment containing the coding sequence for GCaMP6s (53) and a portion of the Internal Ribosome Entry Site (IRES2) sequence was subsequently cloned into the vector, which included the other half of the IRES2 sequence, by restriction/ligation using EcoRI and AvrII enzymes.

found (Student's *t*-test, +100 mV, $p = 0.813$; −100 mV, $p = 0.284$). (*I*) Cartoon of the promoterless plasmid that we used to express GCaMP6s and TRPV3 in iCasp9 landing pad cells (52) (*top panel*) and of the genomic landing pad in iCasp9 cells before (*middle panel*) and after (*bottom panel*) recombination. (*J*) Time course of the current response to 2-APB measured from inside-out patches obtained from iCasp9 cells stably expressing WT TRPV3 (*light blue*) or Halo-TRPV3 (*gray*) channels. Currents were elicited by stepping membrane voltage between −100 (triangles) and +100 mV (circles) for 250 ms, from a holding potential of 0 mV. Currents were normalized to the maximal value measured at 100 mV in each experiment. The black horizontal dotted and continuous lines denote the exposure to each concentration of 2-APB. Data are shown as mean ± SEM ($n = 4$). (*K*) Representative current time course from an inside-out patch expressing Halo-TRPV3, elicited by voltage steps as in (*J*) while the temperature was changed as indicated by the red trace on the top panel. The dashed red line denotes the zero-current level. (*L, M*) Time course of the current response to heat measured from inside-out patches expressing WT TRPV3 (*light blue*) or Halo-TRPV3 (*gray*) channels, measured at +100 mV (L, circles) or −100 mV (M, triangles) using the same voltage-step protocol as in (*J*). The top panel displays the temperature recorded during each experiment. Currents were normalized to the mean value measured at room temperature and 100 mV. Data are shown as the mean ± SEM (WT, $n = 6$; Halo-TRPV3, $n = 12$).

The final vector uses an IRES2 sequence for the co-expression of both GCaMP6s and the mTRPV3 channel with a four amino acid insertion on its N-terminus (M-A-T-T). Amino acid numbering used for TRPV3 throughout the manuscript disregards this insertion. The pCAG-NLS-HA-Bxb1 plasmid was purchased from Addgene (plasmid #51271).

The general site for insertion of the circularly permuted HaloTag (referred to as HaloTag here) (49) into the mouse TRPV3 channel, within the extracellular loop between transmembrane helices S5 and S6, was determined based on TRPV1exCellHalo, a TRPV1 construct with an extracellular cpHalo insertion that can be successfully festooned with Halo dyes (50). We selected an insertion site between S613 and K614 due to the flexibility in this region inferred from structural data, as well as due to the low sequence conservation between TRPV channels in this specific region. To clone the cpHalo insert at the aforementioned insertion site of TRPV3, we used extension overlap PCR, followed by restriction-ligation cloning into our attB_GcaMP6s_IRES2_TRPV3 WT vector using NsiI-HF and NheI-HF (New England Biolabs). Briefly, we amplified the cpHalo sequence using the TRPV1exCellHalo template (50) and primers that annealed to either end of its coding sequence as well as to the downstream and upstream sequence at the insertion site in TRPV3 (P1_fwd, CTGATTGAGAAGTGCTCCTCTGCCTTTGCCCGC; P2_rev, GAACTGC AGTCCTTTTTGTCCTTCCCGGCAAATTCTGGC). Two additional fragments were amplified with reverse-complement primers of P1 or P2 (P1_rev, GCGGGCAAAGGCAGAGGAGCACTTCTCAATCAG; P2_fwd, GCCAG AATTTGCCGGGAAGGACAAAAAGGACTGCAGTTC) and flanking forward or reverse primers (F1_fwd, TGGCCCTGACACACAAAATGAG; F2_rev, CTAGGGGAAATCACTCCCAATTAAAGC) upstream or downstream of the NsiI or NheI sites in the TRPV3 sequence, respectively. The cpHalo fragment was then amplified together with the fragment upstream of the insertion site using primers F1_fwd and P2_rev. This was then amplified with the fragment downstream of the insertion site using F1_fwd and F2_rev. The resulting fragment was cloned using NsiI-HF and NheI-HF using T4 ligase (New England Biolabs). We deleted amino acids P10-K368 (i.e., Δ359 a.a.) from Halo-TRPV3 by restriction/ligation cloning of a dsDNA fragment (Twist Bioscience) coding for the entire N-terminal portion of the channel flanking the truncated amino acids, using the enzymes HindIII-HF (New England Biolabs) and BspEl (Thermo Fisher). To co-express TMEM79 together with GCaMP6s and mTRPV3, we cloned into the vector a dsDNA fragment (Twist Bioscience) that included the C-terminal end of TRPV3, a T2A peptide sequence, and the entire TMEM79 coding sequence, using restriction/ligation cloning and the enzymes KflI (Thermo Fisher) and NheI-HF (New England Biolabs). The T2A peptide causes ribosome skipping, which yields two separate proteins (TRPV3 and TMEM79) from a singular open reading frame. To generate plasmids attB_EGFP-PH-PLCδ_IRES2_Halo-TRPV3 and attB_EGFP-PH-PLCδ_IRES2_Halo-TRPV3_T2A_TMEM79, a dsDNA fragment (Twist Bioscience) containing a coding sequence for the pleckstrin homology domain from rat phospholipase Cδ with an N-terminal EGFP fusion (EGFP-PH-PLCδ) was cloned into the attB_GCaMP6s_IRES2_Halo-TRPV3 or attB_GCaMP6s_IRES2_Halo-TRPV3_T2A_TMEM79 plasmids by restriction/ligation using EcoRI and SacII because these recognition sites flank the entirety of the GCaMP6s coding sequence in these plasmids. A dsDNA fragment containing the coding sequence of the Lysosomal-Associated Membrane Protein 1 (LAMP1) with a C-terminal EGFP fusion was similarly cloned by restriction/ligation using EcoRI and SacII to generate plasmids attB_LAMP1-EGFP_IRES2_Halo-TRPV3 and attB_LAMP1-EGFP_IRES2_Halo-TRPV3_T2A_TMEM79. All dsDNA fragments used for cloning were preamplified by PCR using primers complementary to the adapter sequences on either end of the fragment that are included as part of the Twist Bioscience production process. Ligation reactions were transformed into E. cloni 10G chemically competent cells (Biosearch Technologies). All recombinant DNA vectors were verified by whole-plasmid sequencing (PlasmidSaurus) and/or Sanger sequencing (Eton Bio).

To clone the mouse TRPV3 and mouse TRPV3-Halo coding sequences into the pcDNA1 mammalian cell expression plasmid, the coding sequences for both channel constructs were PCR-amplified from their respective attB plasmids using primers that introduced HindIII (pHindIII_fwd, cagttcctctggaagcttcttgaagac) or NotI (pNotI_rev, gcggccgccagtgtgctacaccgacgtttctgggaac) restriction enzyme recognition sites on either end of the channel coding sequence, which were then used to clone the PCR product into pcDNA by restriction/ligation.

Cell culture and protein expression

Unless specified otherwise, proteins were expressed using HEK293-derived iCasp9 landing pad cells (52), which were a generous gift from Kenneth Matreyek (Case Western Reserve University). Cells were initially maintained under adhesion conditions at 37°C with a 5% CO_2 atmosphere. Adhesion cultures were grown in high-glucose DMEM media supplemented with pyruvate, L-glutamine, phenol red, 10% (v/v) heat-inactivated fetal bovine serum, 10 μg/mL gentamicin, and 2 μg/mL doxycycline. Recombination of the promoterless expression plasmids into the landing pad was carried out as follows: on the first day, iCasp9 cells were seeded onto six-well plates pretreated with 6 μg/mL bovine plasma fibronectin (Sigma) prepared in phosphate-buffered saline (PBS), transfected with the Bxb1 plasmid using FuGENE HD (Promega) following the manufacturer's instructions, and kept in complete media without doxycycline. On the second, third, and fourth day, respectively, cells were transfected again with the promoterless plasmids, the growth media were supplemented with doxycycline, and then the media were supplemented with doxycycline and 10 nM AP1903 (MedChemExpress) to kill off unrecombined cells (52). On the fifth day, the cells were washed once with complete media with doxycycline and allowed to recover for 2–3 days. Cells were then transferred to tissue-culture-treated T25 flasks and subsequently placed in suspension growth conditions upon reaching confluency. To achieve this, cells were detached using 0.25% trypsin and resuspended in FreeStyle 293 Expression Medium (Gibco), supplemented with 2% (v/v) heat-inactivated fetal bovine serum, 10 μg/mL gentamicin, and 2 μg/mL doxycycline. After resuspension, the cells were washed once with suspension growth media and then transferred to 125-mL baffled-bottom glass flasks. The cell densities in the suspension cultures were maintained between 0.5 and 2×10^6 cells/mL. Cells were resuspended and passaged into a new flask with fresh media every other day to minimize cell aggregates as well as cell accumulation on the rim below the liquid/air interface. These cultures were maintained at 37°C with an 8% CO_2 atmosphere and with constant shaking at 135 rpm using a Celltron shaker (Infors HT). A Countess II FL (Thermo Fisher) with a GFP filter cube was used to count cells during culture. Working stock solutions of AP1903 in DMSO were prepared at 10 μM from an initial stock solution of 10 mM. The doxycycline working stock was prepared at a concentration of 2 mg/mL using sterile water. The epifluorescence microscopy and flow cytometry of fluorescently labeled cells as well as the excised patch electrophysiology data included in the paper come from one stable cell line generated via recombination for each construct, kept under suspension growth conditions: WT (no Halo), Halo-TRPV3, Halo-TRPV3 Δ359, and Halo-TRPV3 + TMEM79. The confocal microscopy data and flow cytometry of channel activity in response to 2-APB data were obtained using two additional independently recombined stable cell lines for each of the constructs.

Whole-cell patch-clamp experiments were carried out using transiently transfected HEK293 cells (ATCC, CRL-1573). Cells were kept at 37°C with 5% CO_2 and grown in DMEM with high glucose, pyruvate, L-glutamine, and phenol red, supplemented with 10% fetal bovine serum (vol/vol) and 10 mg mL^{-1} gentamicin. For transient transfection, cells were detached with trypsin, resuspended in DMEM, and seeded onto 3-mL dishes with no. 1 glass coverslips. Transfections were performed immediately after seeding using FuGENE HD (Promega), following manufacturer's instructions. Channel constructs (pcDNA1_TRPV3 or pcDNA1_Halo-TRPV3) were co-transfected with pGreen-Lantern (Invitrogen) at a ratio of 2:1. Patch-clamp recordings were done 18 to 36 h after transfection.

Patch-clamp electrophysiology: Whole-cell recordings

Data were acquired at 5 kHz, filtered at 1 kHz, and digitized using a dPatch amplifier and SutterPatch software (Sutter Instrument). Pipettes were pulled from borosilicate glass (1.5 mm OD x 0.86 mm ID x 75 mm L; Harvard Apparatus) using a P-97 puller (Sutter Instrument) and heat-polished to final resistances between 0.2 and 1.0 MΩ using a MF-200 microforge (World Precision Instruments). An agar bridge (1 M KCl; 4% w/v agar; Teflon tubing) was used to connect the ground electrode chamber and the main recording chamber. Recordings were carried out in a Delta T imaging chamber (Bioptechs). To measure the response of channels to 2-aminoethoxydiphenyl borate (2-APB), we used a gravity-fed RSC-200 perfusion system (BioLogic), in which a rotating motor rapidly switched between different glass capillary sewer pipe solution outlets to face the patch pipette, allowing rapid exchange between solutions. Cells were lifted from the coverslip after giga-seal formation and placed in front of the glass capillary sewer pipe containing control solution before starting the experiment. The bath solution consisted of 130 mM NaCl, 10 mM HEPES, 10 mM EGTA, and 40 mM sucrose (pH 7.4). This same solution supplemented with 10 mM $MgCl_2$ was used in the pipette. The 2-APB stock was prepared fresh each day of recording at a concentration of 1 M in DMSO.

Current-voltage relations were obtained by stepping the voltage from −100 to +120 mV in 20-mV increments. Test pulses had a duration of 400 ms. Voltage was held at −60 mV for 200 ms before and after the test pulses, and a holding potential of 0 mV was used otherwise. The interpulse interval was set to 1.2 s. The mean current-voltage relations were constructed after normalizing data from individual experiments to the steady-state current magnitude recorded at +120 mV. To obtain concentration-response relations to 2-APB, voltage was stepped between −100 and +100 mV. Test pulses had a duration of 500 ms, and the holding potential was 0 mV. The interpulse interval was 1.8 s. Patches were exposed to each 2-APB concentration for the same duration time, with a short exposure to control solution without 2-APB between each stimulation interval. To quantify the concentration-response relations, the peak current magnitude was quantified at each 2-APB exposure interval and normalized to the current magnitude measured in the presence of either 250 μM 2-APB (currents measured at +100 mV) or 1 mM 2-APB (currents measured at −100 mV). Concentration-response relations from individual experiments were fit with the Hill equation using only the datapoints recorded before the onset of desensitization. A Student's *t*-test was applied to test for statistically significant differences between the $K_{1/2}$ values obtained from the Hill fits for WT TRPV3 and Halo-TRPV3. All analysis was carried out using Igor Pro 9 (Wavemetrics) or Microsoft Excel.

Patch-clamp electrophysiology: Inside-out recordings

All recordings using stable cell lines were done in inside-out patches because TRPV3-mediated currents in the whole-cell configuration were too large to maintain adequate voltage clamp. Pipettes were heat-polished to final resistances between 1.0 and 6.0 MΩ. Recordings were performed using a temperature-controlled Delta T imaging chamber (Bioptechs). The Delta T system enables uniform and rapid temperature changes by delivering heat directly from the cover glass floor of the chamber, which has an electrically conductive coating on the underside opposite to where cells are located. We recorded temperature in all experiments using a small thermistor probe placed a few millimeters away from the patch pipette tip, which was lifted from the bottom of the chamber upon giga-seal formation. To measure the response of channels to temperature, a setpoint of 44°C was selected on the Delta T controller starting from room temperature, which caused the temperature to increase from 20°C to 42°C within a few minutes, after which the temperature setpoint was maintained for the rest of the experiment. In experiments involving repeated cycles of heating and cooling, a metal "cooling ring" (Bioptechs) tube shaped into a loop that fits within the Delta T recording chamber was used to rapidly reduce temperature by flowing ice water through the ring. To increase temperature, the ice water flow through the ring was stopped, and the Delta T heat shock function was used in which a strong voltage is applied to the bottom of the chamber. To measure the response of channels to 2-aminoethoxydiphenyl borate (2-APB), we used the gravity-fed RSC-200 perfusion system (BioLogic).

Both the bath and pipette solutions consisted of 130 mM NaCl, 10 mM HEPES, 10 mM EGTA, and 40 mM sucrose (pH 7.4). Currents were elicited by stepping voltage between −100 and 100 mV for 250 ms at each voltage from a holding potential of 0 mV, with an interpulse interval of 3 s. Currents in response to 2-APB were normalized to the maximal steady-state mean current value recorded at 100 mV during each experiment, whereas currents in response to temperature were normalized to the mean steady-state current recorded at room temperature (21°C–22°C) and 100 mV. Normalized current time courses in response to 2-APB were averaged after aligning all experiments along the time axis by the start of the exposure to the agonist. Normalized current time courses in response to temperature were averaged after aligning the currents according to the time course of the recorded temperature. All analysis was carried out using Igor Pro 9 (Wavemetrics) or Microsoft Excel.

Confocal microscopy

Cells for imaging were labeled with JF635i, JF549, and DAPI. The labeling solutions for the membrane-impermeable dye JF635i (Promega and HHMI's Janelia Research Campus) were prepared at 400 nM in extracellular solution consisting of 130 mM NaCl, 10 mM HEPES, 4 mM KCl, 10 mM glucose, 40 mM sucrose, and 0.2 mM $CaCl_2$ at a pH of 7.4. The labeling solutions for the membrane-permeable dye JF549 (Promega) were prepared at 200 nM in the same extracellular solution from a 200 μM dye stock solution in DMSO, prepared following the manufacturer's instructions. DAPI (Millipore Sigma) stock solution at a concentration of 5 mM was prepared in water, and a working concentration of 5 μM was used, prepared in the extracellular solution described above. A 32% paraformaldehyde (PFA) stock solution (15714-S, Electron Microscopy Sciences) was used to prepare 1% PFA working solution in PBS.

Cells were independently seeded, labeled, and imaged in triplicate. A total of 4×10^5 cells per well were seeded on 24-well glass-bottom imaging plates (Corning) pretreated with 6 μg/mL bovine plasma fibronectin in PBS and incubated for 30 min to allow attachment. For live-cell experiments, cells were then labeled for 30 min at 37°C with JF635i. All wells were then washed with extracellular solution and labeled with JF549 for an additional 30 min. Wells were washed and then cells were labeled with DAPI for 5 min. Wells were washed and cells imaged in extracellular solution. For fixed-cell experiments, cells attached to the bottom of fibronectin-treated 24-well plates were washed with PBS and exposed to ice-cold 1% PFA solution on ice for 5 min, followed by thorough washing using PBS. The same labeling procedure as for the live cells was then used. Notably, we compared alternative cell fixation conditions (not shown), evaluating which ones yielded high-quality images of cells without causing significant membrane permeabilization (evaluated using nuclear DAPI fluorescence). We found that glutaraldehyde yielded the highest quality images of cells without membrane permeabilization but also introduced significant autofluorescence over the same spectral range used to image JF549. Ice-cold methanol produced robust membrane permeabilization, and 5% PFA applied for 2 or 5 min seemed to damage cells more than the 1% concentration that we used.

Cells were imaged at room temperature using an AxioObserver Z1 confocal microscope (Zeiss) and a 63× oil-immersion objective (Plan-Apochromat N.A. 1.4). Laser intensity and photomultiplier gain were initially adjusted to avoid pixel saturation on any acquired image at each of the four channels that we acquired: DAPI, Alexa Fluor488, Alexa Fluor 546, and Alexa Fluor 635. The green fluorescence (GCaMP6s, LAMP1, or EGFP-PH-PLCδ) was used to select cells for imaging and for focusing. Images of central cross sections of cells were acquired using fourfold averaging. One to two images per well were acquired for all constructs in

triplicate, using the same imaging settings. All image analysis was carried out on ImageJ (54). After acquisition, images of each of the constructs (WT, Halo-TRPV3, Δ359 a.a., or HaloTRPV3 + TMEM79) were grouped by fluorescence channel, and the lookup tables were normalized. Linear regions of interest (ROIs) drawn across three cells per channel construct were used to obtain linear fluorescence intensity profiles for JF635i and JF549. Figures were generated using Igor Pro 9.

Epifluorescence microscopy

Live cells seeded on fibronectin-treated glass-bottom 24-well plates were labeled with both JF635i and JF549 (dual labeling) or with just one of the two dyes (single label), as described for the confocal experiments except that no DAPI was used. In addition, labeling was carried out using FluoroBrite DMEM (Gibco). Both dual- and single-label cells were prepared together, whereby single-label cells were incubated for 30 min in FluoroBrite DMEM (Gibco) in the absence of dye while the other samples were labeled. Dually labeled cells were independently seeded, labeled, and imaged in triplicate. Only one experiment was carried out for the single-label cells.

Samples were imaged at room temperature on a Nikon Eclipse Ti2 epifluorescence microscope equipped with a motorized stage, a SOLA v-nIR Light Engine Gen-III light source (Lumencor), and an ORCA-Fusion Gen-III sCMOS camera (Hamamatsu Photonics). One set of multichannel images was acquired per well of labeled cells using a 10× objective (CFI60 Plan Apochromat Lambda, NA 0.45, WD 4 mm). Imaged channels consisted of bright-field, FITC (Ex. 466/40 nm; Em. 525/50 nm; Dichr. 495 nm; Semrock Brightline), JF549 (Ex. 575/25 nm; Em. 615/24 nm; Dichr. 596 nm; Semrock Brightline), and JF635i (Ex. 661/20 nm; Em. 732/68 nm; Dichr. 695 nm; Semrock Brightline). For analysis, 20 individual GCaMP6s$^+$ cells per image were randomly selected, and their mean fluorescence intensity in the acquired fluorescent channels was extracted by drawing ROIs around them using the auto-detect tool of NIS Elements (Nikon). The mean fluorescence intensity of the background was obtained by shifting each of the ROIs to a region that did not contain any cells and was subtracted from the mean fluorescence intensity measured from each cell. Cells that had a negative fluorescence intensity after background subtraction were assigned a subtracted fluorescence value of 1. Data analysis and display was carried out using Igor Pro 9. Statistical significance was evaluated using Tukey's HSD multiple comparison test implemented on Igor Pro 9.

Flow cytometry

Cells for flow cytometry were labeled with both JF635i and JF549 (dual labeling) or with just one of the two dyes (single label). Dually labeled cells were independently labeled and analyzed in triplicate. Only one experiment was carried out for the single-label cells. The labeling solutions for JF635i were prepared at 400 nM, unless otherwise indicated, in extracellular solution consisting of 130 mM NaCl, 10 mM HEPES, 4 mM KCl, 10 mM glucose, 40 mM sucrose, and 0.2 mM $CaCl_2$ at a pH of 7.4. The labeling solutions for JF549 were prepared at 200 nM in extracellular solution from a 200 μM stock solution in DMSO. Aliquots of 2.5×10^6 cells per sample were pelleted at $1450 \times g$ and washed with extracellular solution before labeling with JF635i for 30 min at 37°C (dually labeled cells and JF635 single-label cells). Cells were then washed with extracellular solution and labeled with JF549 for 30 min at 37°C (dually labeled cells and JF549 single-label cells). Cells were washed once and resuspended in extracellular solution at a density of 5×10^6 cells/mL before analysis on a BD FACS Aria Fusion SORP Cell Sorter (BD Biosciences), carried out at room temperature at the Center for Biomedical Research Support Microscopy and Flow Cytometry facility at UT Austin. Single live cells were gated based on their forward and side scatter, and their GCaMP6s, JF549, and JF635i fluorescence intensity values were measured using the blue, yellow/green, or red lasers for excitation and GFP-A, PE-A, and Alexa Fluor 646-A optic filters, respectively. The resulting fcs files were opened using Floreada software (http://floreada.io) and saved as csv files for further analysis using Igor Pro 9. No statistical tests were carried out with these data sets, as the large number of cells in each sample caused a drastic reduction in the resulting *p*-values regardless of whether the underlying distributions were visibly different or not. Instead, we rely on the effect size that is visible from the data distributions for each of the constructs. We included the 95% confidence interval or the standard error of the mean between the three replicates in some of the plots to better display the effect sizes for each construct.

To assess the response to 2-APB or camphor of the four TRPV3 channel constructs, unlabeled cells were washed and resuspended in extracellular solution at a density of 5 (1×) or 10×10^6 (2×) cells/mL. The 2× cell suspension was supplemented with an equal volume of extracellular solution with a 2× concentration of camphor or 2-APB. A camphor stock solution at a concentration of 0.5 M in DMSO was used, and the 2× camphor solution (20 mM) was incubated at 46°C to allow for full solubilization of the agonist. A total of 10×10^3 cells were analyzed per sample for the camphor experiments, whereas the 2-APB experiments were carried out in triplicate, analyzing 10×10^3 cells per replicate and condition.

Polyacrylamide gel electrophoresis and in-gel fluorescence

Six-well plates pretreated with fibronectin (6 μg/mL in PBS) for 30 min were used. A total of 3×10^6 suspension culture cells were seeded per well and allowed to attach for 30 min at 37°C in extracellular solution (130 mM NaCl, 10 mM HEPES, 4 mM KCl, 10 mM glucose, 40 mM sucrose, 0.2 mM $CaCl_2$, pH 7.4). After attachment, cells were labeled for 30 min at 37°C with JF549 at a concentration of 200 nM in extracellular solution. The dye was washed with extracellular solution, followed by incubation of cells for 10 min at 37°C in a 10 mM EDTA PBS solution. Cells were then lifted and resuspended in PBS, spun down at $1000 \times g$, and lysed using CHAPS lysis buffer (Invitrogen) supplemented with Halt Protease Inhibitor (Thermo Fisher Scientific). During lysis, cells were kept on ice for 30 min and vortexed every 5 min. Lysates were spun down at $10{,}000 \times g$ for 2 min, and a fraction of the lysate supernatant was supplemented with NuPAGE 10× sample reducing agent (Invitrogen) and NuPAGE 4× LDS sample buffer (Invitrogen), loaded on a NuPAGE 4%–12% Bis-Tris gel (Invitrogen), and run at 200 V using NuPAGE MOPS buffer (Invitrogen). PageRuler Plus molecular weight marker (Invitrogen) and HiMark Pre-Stained Protein Standard ladder (Invitrogen) were also loaded on the gels. Three cell samples (one well in a six-well plate) expressing Halo-TRPV3 + TMEM79 were independently seeded, labeled, and lysed. One replicate was run in a gel together with a sample of Halo-TRPV3 cell lysate labeled with JF549, and the other two replicates were run on a separate gel together with a second independently prepared sample of Halo-TRPV3 cell lysate labeled with JF549. After electrophoresis, gels were imaged on a Typhoon RGB imager (Amersham) using Cy3 settings for JF549 and Cy5 settings for the molecular weight markers. Gels were then Coomassie-stained and imaged using a ChemiDoc imager (Bio-Rad). To quantify the relative protein abundance, gel lane fluorescence intensity profiles were first obtained using the built-in gel analysis tool on ImageJ. The area under the curve corresponding to each of the two Halo-TRPV3 peaks was separately quantified from the fluorescence profiles and normalized to the sum of the area under the two peaks.

RESULTS

An extracellular-facing HaloTag insertion in TRPV3 causes minimal alterations in channel activation by voltage, 2-APB, and heat

To create a fluorescent reporter of TRPV3 channel subcellular localization, we inserted a circularly permuted HaloTag (49) into the extracellular loop connecting transmembrane

helices S5 and S6 of the mouse TRPV3 channel (Fig. 1 *A*). This region is likely flexible and shows limited sequence conservation among closely related TRPV homolog genes (Fig. 1 *B*), raising the possibility that the HaloTag insertion in this region would have a minimal impact on channel function. To determine whether this was the case, we recorded the activity of both WT mouse TRPV3 as well as the Halo-TRPV3 fusion protein using whole-cell patch-clamp recordings of HEK293 cells that were transiently transfected with each construct. We found that currents elicited by depolarizing voltage steps at room temperature in the absence of any agonist had similar kinetics and voltage dependence between the two constructs (Fig. 1, *C* and *D*). When cells expressing WT TRPV3 or Halo-TRPV3 were challenged by increasing concentrations of the TRPV agonist 2-APB, we observed that the two cell groups exhibited similar concentration-response relations of channel activation measured at both a positive or negative membrane potential (Fig. 1, *E–H*). We also observed that exposing cells to the two highest concentrations of 2-APB that we tested (1 and 2 mM) resulted in prominent current desensitization that was more pronounced in cells expressing Halo-TRPV3 than WT TRPV3 channels (Fig. 1, E–*G*). Overall, our findings establish that key properties of TRPV3 channels, such as voltage dependence, kinetics, and sensitivity to activation by 2-APB are unaltered in the Halo-TRPV3 fusion, which otherwise exhibits more pronounced desensitization at extreme 2-APB concentrations than WT channels.

Because a major motivation to generate the Halo-TRPV3 tool is its applications for high-throughput screens of channel activity and cell surface expression, we tested our Halo-based reporter using a HEK293-derived landing pad system (52) that is widely used for the expression and screening of protein variant libraries (55–59). Through recombination-dependent integration of a promoterless plasmid into the genomic landing pad that these cells have (Fig. 1 *I*), we generated a stable cell line expressing the Halo-TRPV3 fusion protein together with the fluorescent calcium reporter GCaMP6s (53), which allows the optical interrogation of calcium-permeable channel activity. To further test that the HaloTag does not interfere with regular channel activity, we performed patch-clamp recordings in inside-out patches excised from landing pad cells expressing WT TRPV3 (no Halo) or Halo-TRPV3 channels (Fig. 1, *J*–M). Exposure of patches to 2-APB, which we applied at a concentration (60 μM) that slowly drives activation of TRPV3 channels (30,60,61), yielded identical responses by WT or Halo-TRPV3 channels (Fig. 1 *J*), which showed perfectly superposed time-courses reflective of the slow activation process at this concentration of 2-APB. This result obtained in excised patches further supports our conclusions using whole-cell recordings, that both channel constructs have the same sensitivity to activation by 2-APB. We also observed that subsequent exposure to a much higher concentration (1 mM) of 2-APB caused Halo-TRPV3 channels to desensitize more rapidly than WT channels (Fig. 1 *J*), consistent with our whole-cell experiments.

Heat and voltage are the only known physiological activators of TRPV3. We therefore tested whether the sensitivity to temperature was altered in Halo-TRPV3 channels. We found that patches expressing Halo-TRPV3 channels showed a slowly sensitizing response to temperature when exposed to repeated cycles of heating and cooling (Fig. 1 *K*), as has been previously described for WT TRPV3 channels (1,6). We compared the responses of both constructs to a sustained heat challenge (40°C–45°C), which should elicit the same activation response as that caused by repeated heating and cooling cycles, and found that WT and Halo-TRPV3 channels exhibit comparable activation at a positive and negative voltage (Fig. 1, *L* and *M*). The overall agreement between the measured currents as a function of both temperature and time for WT TRPV3 and Halo-TRPV3 indicate that the HaloTag insertion maintains temperature sensitivity as well as the slow kinetics that are characteristic of the TRPV3 channel.

Visualizing TRPV3 channel subcellular localization by HaloTag fluorescence labeling and confocal microscopy

The HaloTag insertion on the extracellular surface of the TRPV3 channel should allow us to selectively label channels localized to the cell surface using the membrane-impermeable JF635i dye, whereas subsequent labeling with the membrane-permeable JF549 dye should label only channels within intracellular compartments. To adequately distinguish between the two channel populations in cells, it is necessary to ensure that all extracellularly available sites are saturated by the JF635i dye. We therefore labeled cells with various concentrations of the JF635i dye before labeling with JF549 and analyzed the labeling intensity per cell using flow cytometry (Fig. S1). The fluorescence intensity of GCaMP6s expressed in the cytoplasm of cells allowed us to identify cells expressing Halo-TRPV3 channels independently of the HaloTag, which represented over 70% of the total population of single cells that we analyzed (Fig. S1 *A*). Our measurements show that the labeling ratio between JF635i and JF549 reaches saturation at a JF635i concentration above 250 nM (Fig. S1 *B–G*), so we used a concentration of 400 nM JF635i in the rest of our experiments.

We next tested our Halo-TRPV3 channel labeling strategy to visualize TRPV3 channel subcellular localization in living landing pad cells by confocal microscopy (Fig. 2). To assess the amount of nonspecific labeling by the HaloTag dyes, we included in our experiment cells expressing WT channels lacking a HaloTag (Fig. 2 *A*). We also included landing pad cells stably expressing Halo-TRPV3 channels together with mouse TMEM79 (Fig. S2 *A*), which was shown to retain TRPV3 channels in the

endoplasmic reticulum (ER) and thus reduce their expression in the plasma membrane (9) (Fig. 2 *L*). We also sought a TRPV3 channel variant lacking cell surface expression as negative control to further benchmark our channel labeling and detection strategy; although several TRPV3 channel variants associated with Olmsted syndrome have reported trafficking defects in HaCaT cells (62), these variants still localize to the plasma membrane in HEK293 cells and elicit robust currents (35), making them highly cytotoxic. Otherwise, we did not find any other reports describing TRPV3 variants with defective surface expression. To address this limitation, we engineered a Halo-TRPV3 channel with an N-terminal deletion of 359 amino acids (Halo-TRPV3 Δ359 a.a.) (Fig. 2 *R*) that we assumed would be sufficiently large to disrupt its trafficking to the plasma membrane without affecting the folding of the HaloTag. Notably, similar N-terminal truncations dramatically reduce surface expression of TRPV1 (63) and TRPV2 (64) channels that are structurally similar to TRPV3.

Stable cell lines expressing each of the constructs were maintained under suspension culture conditions; however, cells were still able to readily attach to fibronectin-coated coverslip glass and could be labeled within 30 min after seeding without any considerable amount of cell detachment during the entire labeling process that lasted over an hour. Attached cells were labeled with JF635i, the membrane-impermeable dye, for 30 min, followed by labeling with JF549, the membrane-permeable dye, for another 30 min. Cells were finally labeled with DAPI to identify those that became permeabilized during the labeling process (Fig. 2, *B*, *G*, *M*, and *S*).

As expected, the four TRPV3 constructs used in this study exhibited strong GCaMP6s fluorescence (Fig. 2, *B*, *G*, *M*, and *S*), including cells expressing WT TRPV3 channels without a HaloTag (Fig. 2 *B*). The lack of JF549 and JF635i fluorescence in cells expressing WT TRPV3 channels confirmed the specificity of both fluorescent dye ligands for the HaloTag (Fig. 2, *C–E*). The Halo-TRPV3 construct exhibited robust JF635i labeling at the cell periphery (Fig. 2, *H*, *I*, and *K*), with comparatively lower levels of intracellular labeling observed with JF549 (Fig. 2, *H–J*), aligning with the established localization of TRPV3 in the plasma membrane (Fig. 2 *F*). Linear fluorescence intensity profiles measured across individual cells in confocal microscopy images reveal that the fluorescence intensity of JF635i was higher toward the edges of cells, consistent with a localization in the plasma membrane, whereas JF549 fluorescence showed strong localization toward the cell interior (Fig. 2 *I*). In contrast, the Halo-TRPV3 Δ359 a.a. N-terminal deletion construct exhibited JF549 fluorescence localized intracellularly (Fig. 2, *T–V*), with virtually undetectable JF635i labeling (Fig. 2, *T*, *U*, and *W*), indicating that trafficking to the plasma membrane is largely impaired in this construct (Fig. 2 *R*). Notably, the intracellular distribution of this construct was clearly different from that of Halo-TRPV3 channels. In cells co-expressing Halo-TRPV3 channels and TMEM79 proteins, we observed prominent JF549 fluorescence distributed at the cell edges but also intracellularly (Fig. 2, *N–P*), and weaker JF635i fluorescence localizing at the cell edges where the plasma membrane should be (Fig. 2, *N*, *O*, and *Q*). When compared with Halo-TRPV3, this construct exhibited a reduced JF635i signal and increased JF549 labeling, indicating a partial interference with membrane transport by the co-expression of TMEM79 (Fig. 2, *F–I* and *N–Q*), consistent with previous observations (9).

To co-express the Halo-TRPV3 and TMEM79 in landing pad cells, we introduced a T2A sequence between the coding sequences of the two proteins (Fig. S2 *A*). During protein translation, the T2A peptide causes ribosome skipping, which terminates translation of the first protein while continuing the translation of the second protein in the reading frame. Although this tool is widely used, there is a possibility that the prominent intracellular retention of Halo-TRPV3 channels co-expressed with TMEM79 that we observed could be caused by a failure of the T2A sequence to produce two separate proteins. To test this possibility, we labeled with JF549 cells expressing Halo-TRPV3 with or without TMEM79 and analyzed the total cell lysates from these cells by SDS-PAGE and in-gel fluorescence arising from Halo-TRPV3 tagged with JF549. We were able to detect a prominent JF549 fluorescence band on the gel with a molecular weight consistent with a single Halo-TRPV3 subunit (Fig. S2 *B*) in lysates from both groups of cells. Notably, only the lysate from cells co-expressing TMEM79 exhibited a JF549 fluorescent gel band whose molecular weight agreed with what would be expected for a fusion protein composed of TRPV3 and TMEM79 (Fig. S2 *B*). However, the low fluorescence intensity of this band establishes that this is a minor product of translation, representing about 1% of the total labeled Halo-TRPV3 protein (Fig. S2 *C*). This finding indicates that the T2A sequence in our cells effectively results in the production of the Halo-TRPV3 and TMEM79 as two separate proteins.

One caveat of our labeling strategy is the long incubation period with each of the HaloTag dyes, during which significant membrane remodeling could be occurring in live cells, especially at 37°C. To test whether this represents an issue, we set out to image cells that were fixed before labeling. We found that using 1% paraformaldehyde applied for 5 min did not permeabilize cells, as indicated by the absence of DAPI staining within the nuclei of most cells (Fig. S3 *B*, *H*, and *N*), which was indistinguishable from what we had observed in live cells (Fig. 2, *G*, *M*, and *S*). Keeping the plasma membrane barrier intact in cells after fixation allowed us to take advantage of the membrane-impermeant JF635i for selective labeling of channels in the cell surface of fixed cells. Although we observed slightly sharper JF635i labeling at the cell edges in the fixed cells (Fig. S3) relative to the

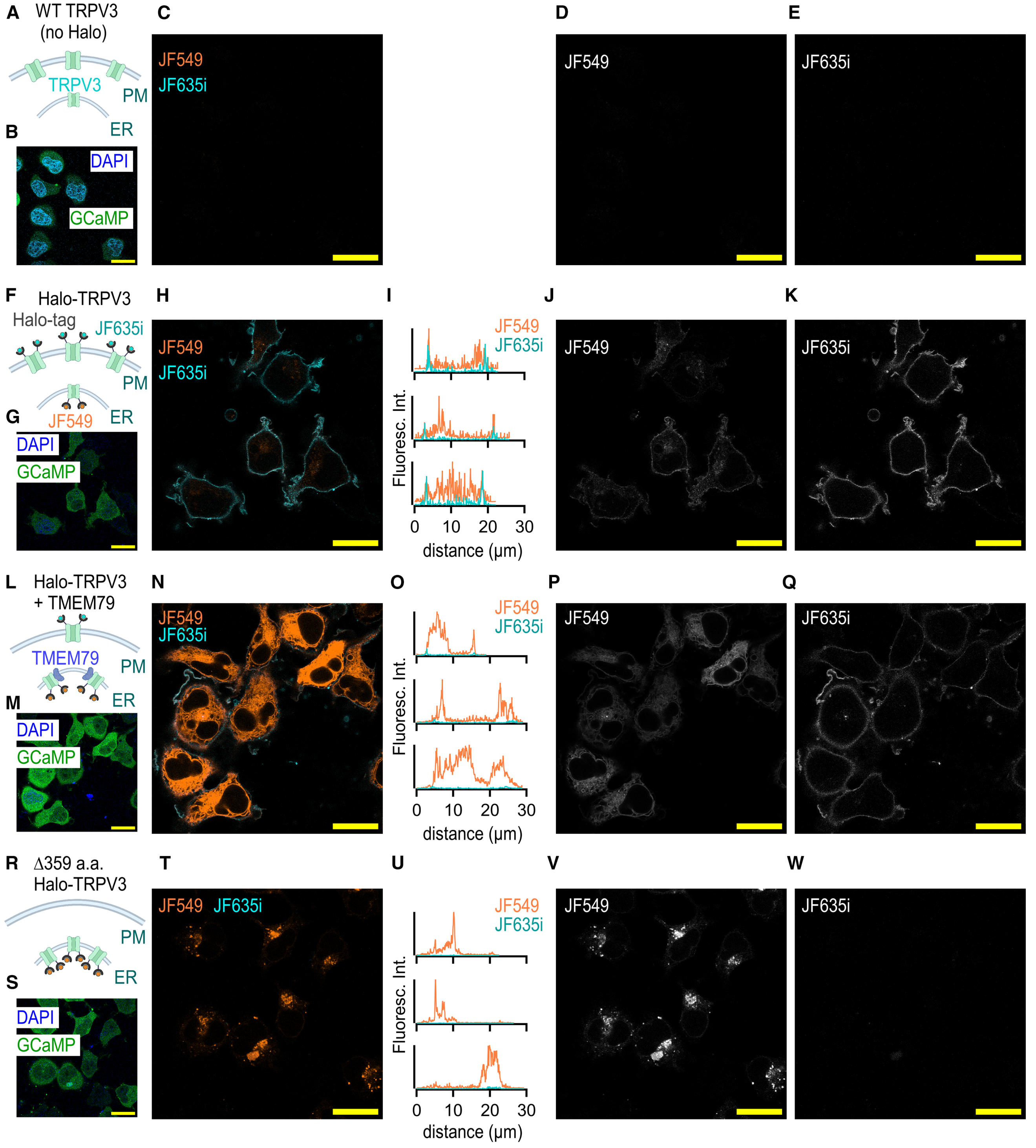

FIGURE 2 Visualization of Halo-TRPV3 subcellular localization by confocal microscopy in live cells. (*A*, *F*, *L*, and *R*) Cartoons depicting subcellular localization of WT TRPV3 (no Halo) (*A*), Halo-TRPV3 (*F*), Halo-TRPV3 + TMEM79 (*L*), and Halo-TRPV3 Δ359 a.a. (*R*) as determined by our labeling approach using a membrane-impermeable dye (*cyan*, JF635i) followed by a membrane-permeable dye (*orange*, JF549). PM stands for plasma membrane and ER for endoplasmic reticulum and other intracellular compartments. (*B*, *G*, *M*, and *S*) Representative GCaMP6s (*green*) and DAPI (*blue*) overlaid confocal microscopy images of live cells labeled with DAPI, JF635i, and JF549 and acquired using a 60× objective. Scale bars represent 20 μm. (*C*, *H*, *N*, and *T*) Representative JF549 (*orange*) and JF635i (*cyan*) overlaid confocal microscopy images of the same fields of view as in (*B*), (*G*), (*M*), and (*S*). (*I*, *O*, and *U*) JF635i (*cyan*) and JF549 (*orange*) fluorescence intensity line profiles from three representative confocal microscopy images of cells expressing Halo-TRPV3 (*I*), Halo-TRPV3 + TMEM79 (*O*), or Halo-TRPV3 Δ359 a.a. (*U*). (*D*, *J*, *P*, and *V*) Representative JF549 confocal microscopy images of the same fields of view as in (*B*), (*G*), (*M*), and (*S*). (*E*, *K*, *Q*, and *W*) Representative JF635i confocal microscopy images of the same fields of view as in (*B*), (*G*), (*M*), and (*S*).

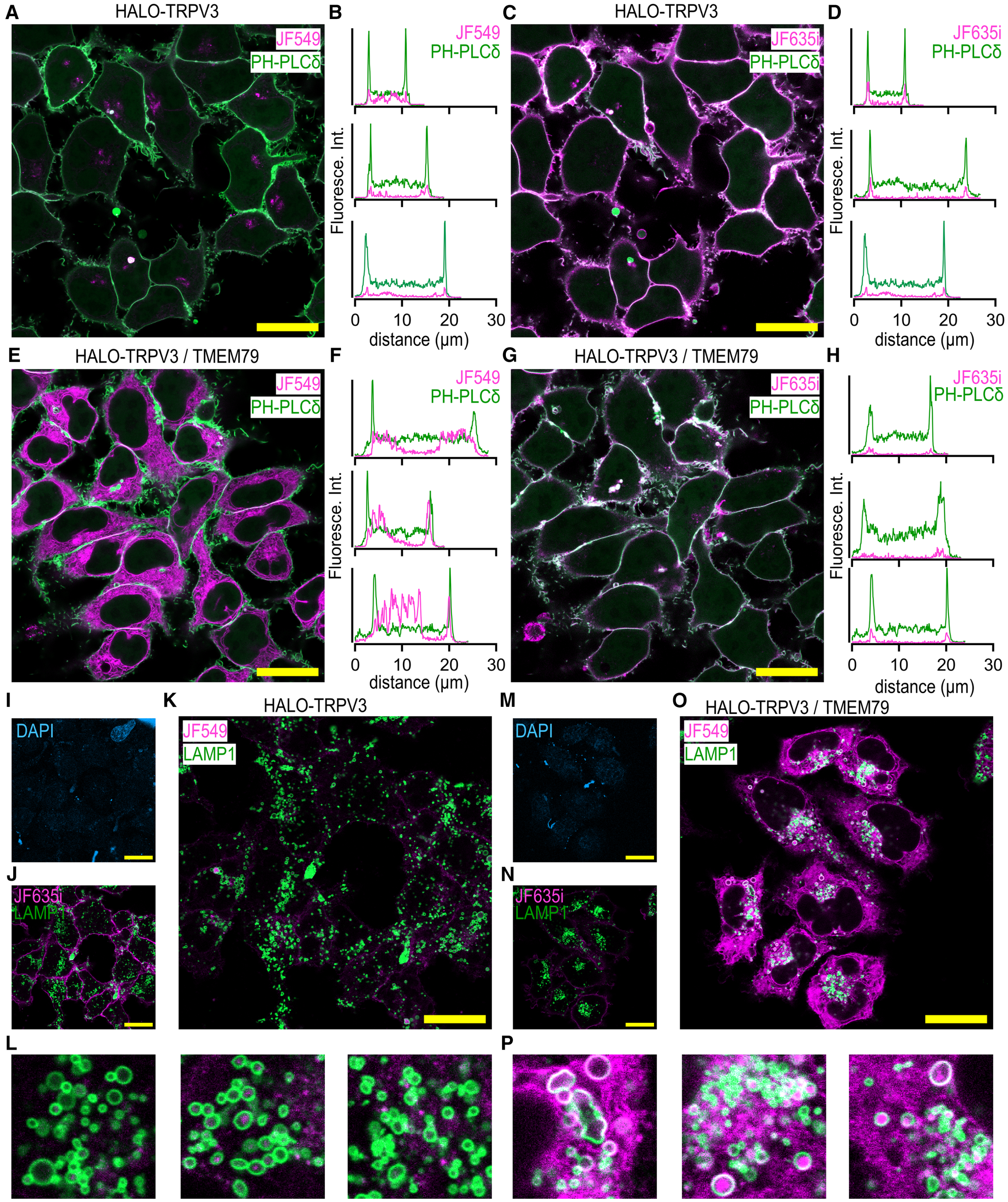

FIGURE 3 Plasma membrane and lysosomal marker co-localization with Halo-TRPV3. (*A* and *E*) Representative EGFP-PH-PLCδ (*green*) and JF549 (*magenta*) overlaid confocal microscopy images of live cells expressing EGFP-PH-PLCδ and Halo-TRPV3 alone (*A*) or Halo-TRPV3 and TMEM79 (*E*), labeled with JF635i, JF549, and DAPI. Images were acquired using a 60× objective. Scale bars represent 20 μm. (*B*, *F*, *D*, and *H*) Fluorescence intensity line profiles for EGFP-PH-PLCδ (*green*), JF549 (B, F *in magenta*), or JF635i (D, H *in magenta*) fluorescence intensity line profiles from three representative confocal microscopy images of cells expressing Halo-TRPV3 (*B*, *D*) or Halo-TRPV3 + TMEM79 (*F*, *H*). (*C*, *G*) Representative EGFP-PH-PLCδ (*green*) and JF635i (*magenta*) overlaid confocal microscopy images of live cells expressing Halo-TRPV3 alone (*C*) or Halo-TRPV3 co-expressed with TMEM79 (*G*). Images are of the same cells as in (*A*) and (*E*). Scale bars represent 20 μm. (*I*, *M*) Representative DAPI confocal microscopy images of live cells expressing Halo-TRPV3 (I) or Halo-TRPV3 and TMEM79 (*M*) and acquired using a 60× objective. Scale bars represent 20 μm. (*J*, *N*) Representative LAMP1 (*green*) and JF635i (*magenta*) overlaid confocal microscopy images of live cells expressing LAMP1 and Halo-TRPV3 (*J*) or Halo-TRPV3 and TMEM79 (*N*) and

(legend continued on next page)

live cells (Fig. 2), all key conclusions about the subcellular localization of Halo-TRPV3 channels that we reached using live cells were consistent with the images that we obtained using fixed cells. This indicates that there are no prominent membrane remodeling processes occurring in the landing pad cells during the live-cell labeling process with the HaloTag dyes.

To more specifically interrogate the subcellular localization of Halo-TRPV3 channels, we generated stable cell lines where instead of GCaMP6s, the Halo-TRPV3 and TMEM79 proteins were co-expressed with either the pleckstrin homology domain from phospholipase Cδ (PH-PLCδ) fused to EGFP (EGFP-PH-PLCδ) (Fig. 3, *A–H*), which resides at the plasma membrane, or with LAMP1-EGFP, which localizes to lysosomes (Fig. 3, *I–P*). Notably, co-expression of TRPV3 with TMEM79 was previously shown to promote its localization and degradation in lysosomes (9). Using live cells labeled as before, we observed extensive co-localization between the plasma membrane marker EGFP-PH-PLCδ and JF635i-labeled Halo-TRPV3 channels in cells with or without TMEM79 (Fig. 3, *A–H*), consistent with selective labeling of surface-expressed channels with JF635i. Labeling of lysosomes revealed that some but not all lysosomes appeared to contain JF549-labeled Halo-TRPV3 channels in cells expressing TMEM79 (Fig. 3, *O and P*), whereas none of the cells we imaged that expressed only LAMP1-EGFP and Halo-TRPV3 exhibited co-localization between these two markers (Fig. 3, *K* and *L*).

Quantitation of relative Halo-TRPV3 abundance between the cell surface and intracellular compartments by epifluorescence microscopy in live cells

The specificity of our approach to label channels in the plasma membrane separately from those in intracellular compartments has the potential to enable quantitative analysis of subcellular channel localization using techniques like epifluorescence or flow cytometry that have limited spatial resolution but that are compatible with high-throughput screens and rapid analysis and quantitation procedures. We next evaluated whether the alterations in subcellular localization that were made evident by confocal microscopy in cells expressing GCaMP6s together with Halo-TRPV3, Halo-TRPV3 Δ359 a.a., or Halo-TRPV3 + TMEM79 could be quantified using widefield epifluorescence microscopy at low magnification. To do this, we first labeled channels in the cell surface with the membrane-impermeable dye JF635i and then labeled channels in intracellular compartments with JF549 using the same cell seeding and labeling protocol as for the confocal microscopy experiments, except that no DAPI was included. We seeded, labeled, and imaged three replicates per test group using a 10× objective (Fig. S4), and then we picked for analysis 20 individual cells per replicate, which were randomly selected solely based on their positive expression of GCaMP6s. We display the JF549 and JF635i fluorescence intensity distributions of the analyzed cells directly using violin plots (Fig. 4, *A* and *B*) and on a logarithmic scale using histograms (Fig. 4, *C* and *D*).

The fluorescence labeling intensity distributions in each of the test groups showed solid qualitative agreement with what we had seen on the confocal microscopy images: 1) cells from the four test groups expressed similar levels of GCaMP6s (Fig. S4 *A*). 2) Labeling by JF549 and JF635i in iCasp9 landing pad cells is highly specific to proteins that contain a HaloTag, because the labeling intensity of WT TRPV3 channels without a HaloTag was negligible (Figs. 4 and S4). 3) Halo-TRPV3 shows the most robust localization in the plasma membrane, with statistically significant differences in its labeling intensity by JF635i relative to all other constructs (Figs. 4, *B–D*; S4 *C*). 4) When co-expressed with TMEM79, Halo-TRPV3 shows more pronounced localization in intracellular compartments that is significantly different than the other test groups (Figs. 4, *A–C*; S4 *B*). This is consistent with its described effect of retaining TRPV3 channels in the ER and driving them into lysosomes (9). 5) The Halo-TRPV3 Δ359 a.a. truncated channel is not trafficked to the plasma membrane, because its labeling intensity by JF635i shows no statistically significant differences relative to cells expressing WT TRPV3 without a HaloTag, whereas also being significantly different from cells that express full-length Halo-TRPV3 (Figs. 4, *B–D*; S4 *C*). Importantly, this construct is expressed but it accumulates in intracellular compartments at levels similar to those of Halo-TRPV3, as indicated by its robust labeling with JF549 (Figs. 4, *A–C*; S4 *B*), except that Δ359 a.a. channels appear to concentrate in a narrower space inside the cells compared with full-length channels (Fig. S4B).

We also analyzed cells that were single labeled with either JF549 (Fig. S5 *A* and *B*) or JF635i (Fig. S5 *C* and *D*) and obtained results that were quantitatively similar to those from dually labeled cells, except that that JF549 labeling was similar between single-label cells expressing Halo-TRPV3 alone or with TMEM79 (Fig. S5 *A*), in contrast with the lower JF549 labeling that we observed for dual-labeled cells in the absence of TMEM79 (Fig. 4, *A* and *C*). This observation indicates that co-expression of TMEM79 does

acquired using a 60× objective. Images are of the same cells that are in (*I*) or (*M*). Scale bars represent 20 μm. (*K*, *O*) Representative LAMP1 (*green*) and JF549 (*magenta*) overlaid confocal microscopy images of live cells expressing LAMP1 and Halo-TRPV3 (*K*) or Halo-TRPV3 and TMEM79 (*O*). Images are of the same cells that are in (*J*) or (*N*). Scale bars represent 20 μm. (*L*, *P*) Magnified views of the images in (*K*) and (*O*), with overlaid JF549 and LAMP1 fluorescence. Each image is 9 μm × 9 μm in size.

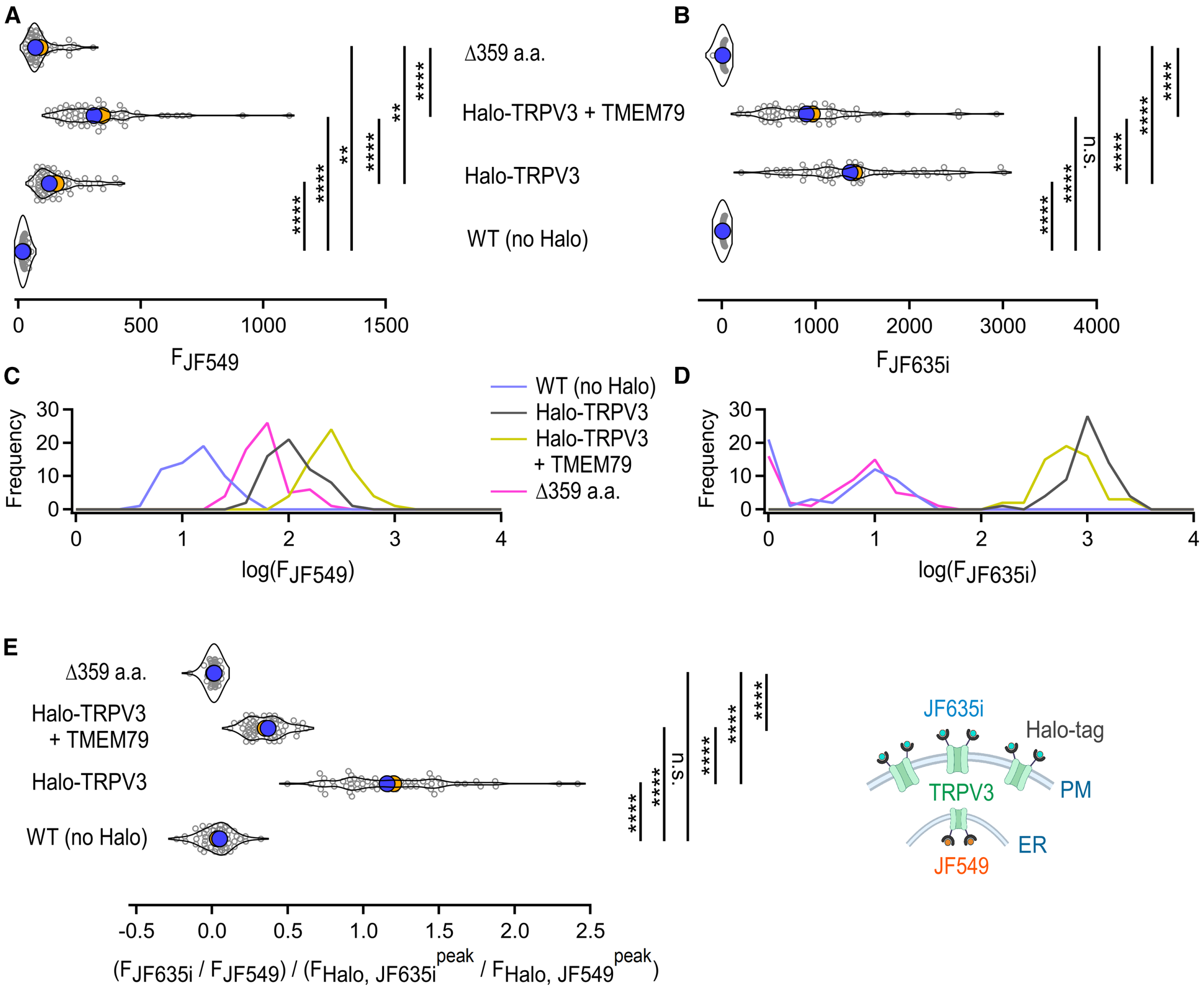

FIGURE 4 Quantitation of surface-expressed vs. intracellular Halo-TRPV3 channels by epifluorescence microscopy. (*A*) Violin plots of the mean JF549 fluorescence intensity of individual cells measured from epifluorescence microscopy images as in Fig. S4 *B* of cells labeled with both JF635i and JF549. Plots contain aggregate data from three replicate experiments, with 20 cells per replicate randomly chosen based on the presence of GCaMP6s fluorescence (Fig. S4 *A*). Data for individual cells are shown as open gray circles, and the mean and median are shown as yellow and blue circles, respectively. Statistical significance was evaluated using a Tukey HSD test (Halo vs. WT, $p = 5.05 \times 10^{-10}$; Halo vs. Δ359, $p = 0.0035$; Halo vs. TMEM79, $p = 6 \times 10^{-13}$; TMEM79 vs. WT, $p = 0$; TMEM79 vs. Δ359, $p = 0$; WT vs. Δ359, $p = 0.0023$). (*B*) Violin plots of the mean JF636i fluorescence intensity of the same cells as in (A). Data are depicted as in (*A*). Statistical significance was evaluated using a Tukey HSD test (Halo vs. WT, $p = 0$; Halo vs. Δ359, $p = 0$; Halo vs. TMEM79, $p = 7.78 \times 10^{-8}$; TMEM79 vs. WT, $p = 0$; TMEM79 vs. Δ359, $p = 0$; WT vs. Δ359, $p = 1$). (*C*) Histograms showing the mean JF549 fluorescence intensity distributions for the data depicted in (*A*). (*D*) Histograms showing the mean JF635i fluorescence intensity distributions for the data depicted in (*B*). (*E*) Violin plots of the subcellular localization ratio between data in (*A*) and (*B*), normalized by the ratio between the peaks of the JF635i and JF549 fluorescence distribution histograms for cells expressing Halo-TRPV3. Statistical significance was evaluated using a Tukey HSD test (Halo vs. WT, $p = 0$; Halo vs. Δs359, $p = 0$; Halo vs. TMEM79, $p = 0$; TMEM79 vs. WT, $p = 2.40 \times 10^{-11}$; TMEM79 vs. Δ359, $p = 4.93 \times 10^{-12}$; WT vs. Δ359, $p = 0.909$).

not reduce the overall abundance of Halo-TRPV3 channels in the cell, which is reflected in the total JF549 labeling intensity in single-label cells, but rather increases the fraction of intracellularly localized channels relative to those in the plasma membrane.

Although each of the conclusions described above are based on statistically significant differences in the labeling intensity distributions of individual dyes between test groups, we realize that some of those differences are not strikingly evident by eye. Assuming that Halo-TRPV3 channel transcription and translation follow a similar rate across the different cell groups, a protein with trafficking defects would not only show decreased expression in the plasma membrane but also accumulation in intracellular compartments. This is indeed what we observe in cells expressing both Halo-TRPV3 and TMEM79. Thus, the ratio between the fluorescence labeling intensity by JF635i and JF549 (Fig. 4 *E*), which we call the subcellular localization ratio, provides a more sensitive metric to assess trafficking perturbations that affect surface expression of Halo-TRPV3 channels. This subcellular localization ratio could be particularly helpful in certain types of high-throughput screens that suffer

from poor signal/noise ratios, like deep mutational scanning. Together, our findings confirm that the HaloTag does not impair TRPV3 membrane trafficking, further supporting its use in subcellular localization studies. Further, we have established that this tool can be readily used to obtain information about the subcellular localization of these channels at the nanoscale when using confocal microscopy, or with limited spatial resolution but increased throughput when using epifluorescence.

Quantitation of relative Halo-TRPV3 abundance between the cell surface and intracellular compartments by flow cytometry in live cells

Flow cytometry can provide the highest level of throughput for analyzing the fluorescence labeling intensity of single cells. Consequently, fluorescence-activated cell sorting (FACS) is utilized in combination with next-generation sequencing to screen protein variant libraries large enough to contain all possible single-residue variants of a protein like TRPV3 and to obtain information about the phenotype of each of the variants in the library (65,66). The Halo-TRPV3 tool that we have generated could be ideal to carry out a mutational screen to comprehensively identify which amino acid positions in the channel are important for its subcellular localization as well as its activity as an ion channel. We therefore set out to use flow cytometry to analyze the fluorescence labeling intensity of Halo-TRPV3 channels, as well as Δ359 a.a. or Halo-TRPV3 co-expressed with TMEM79. In this case, cells were kept in suspension during labeling with JF635i and JF549. Importantly, growth in suspension facilitated cell resuspension before analysis by flow cytometry and would make it simpler to scale up the size of the culture for performing cell sorting experiments involving large libraries.

In the flow cytometer, single, live cells were selected by their forward and side scattering, and fluorescence intensity measurements for JF549 and JF635i (Fig. 5 *A*) were acquired only for cells that expressed GCaMP6s (Fig. 5 *B*). Analysis of dually labeled cells using flow cytometry revealed relatively narrow and mostly single-component distributions for each of the four groups of cells based on the fluorescence intensity of GCaMP6s, JF549, or JF635i (Fig. 5, *A* and *B*), indicating that subcellular channel localization and labeling in iCasp9 landing pad cells is satisfactorily homogenous. When viewed as logarithmic histograms of the fluorescence intensity distribution for each individual dye in cells, the same conclusions can be reached as those obtained with epifluorescence regarding the specificity of the labeling for Halo-containing proteins relative to WT TRPV3 (no Halo), as well as the negative effects on trafficking caused by the N-terminal truncation in Halo-TRPV3 Δ359 a.a. or by the co-expression with TMEM79 (Fig. 5 *A*, top and right panels). However, the robust differences between the subcellular localization of Halo-TRPV3 channels in the three cells groups can be best observed when grouping cells based on their labeling intensity by both dyes, which we show as a two-dimensional plot in the center panel of Fig. 5 *A* or as a logarithmic subcellular localization ratio in Fig. 5 *E*. The differences in effect size between the three groups of cells are made abundantly clear in these plots, establishing that flow cytometry is indeed an ideal way to exploit the capabilities of the Halo-TRPV3 tool to learn about the sequence determinants of TRPV3 channel subcellular localization.

To facilitate quantitative comparison between Halo-TRPV3 relative to the other three test groups in terms of labeling with each dye, we generated plots where the mean of the nonspecific labeling intensity (assessed from cells expressing WT TRPV3) was subtracted from the fluorescence intensities values of each individual cell, followed by normalization by the mean fluorescence intensity of Halo-TRPV3 channels (Fig. 5, *C* and *D*). These plots are consistent with our observations using microscopy and further underscore the conclusion that the major effect of TMEM79 is to increase intracellular Halo-TRPV3 channel localization rather than a decrease in its expression on the cell surface. When analyzing cells that were singly labeled with either JF549 (Fig. S6 *A*) or JF635i (Fig, S6 *B*), we also observed results that are consistent with the quantitation obtained from epifluorescence microscopy images. Importantly, we find that cells expressing Halo-TRPV3 with or without TMEM79 show similar labeling intensity by JF549, further supporting our conclusion that TMEM79 has the effect of shifting the localization of Halo-TRPV3 toward intracellular compartments without largely decreasing total protein abundance. Notably, the epifluorescence images as well as the flow cytometry analysis establish that there is satisfactory spectral separation between GCaMP6s, JF549, and JF635i to separately analyze the fluorescence intensity arising from each of these fluorophores, in addition to the DAPI that was used in our confocal microscopy experiments.

Finally, we determined if GCaMP6s can be utilized to assess channel activity in response to activators, because TRPV3 channel activation is expected to result in calcium entry into the cytoplasm of cells . We analyzed unlabeled cells expressing Halo-TRPV3 in the presence or absence of various concentrations of 2-APB. We included Halo-TRPV3 Δ359 a.a. as a negative control, because our labeling approach indicates that this construct is not trafficked to the plasma membrane. The inclusion of this control is important because 2-APB is known to have effects on many other types of ion channels that could be endogenously expressed in the HEK293-derived iCasp9 landing pad cells. We found that cytoplasmic Ca^{2+} as evaluated by the fluorescence intensity of GCaMP6s in cells expressing Halo-TRPV3 channels increased as a function of the concentration of 2-APB, whereas cells expressing Halo-TRPV3 Δ359 a.a. lacked responses to the agonist

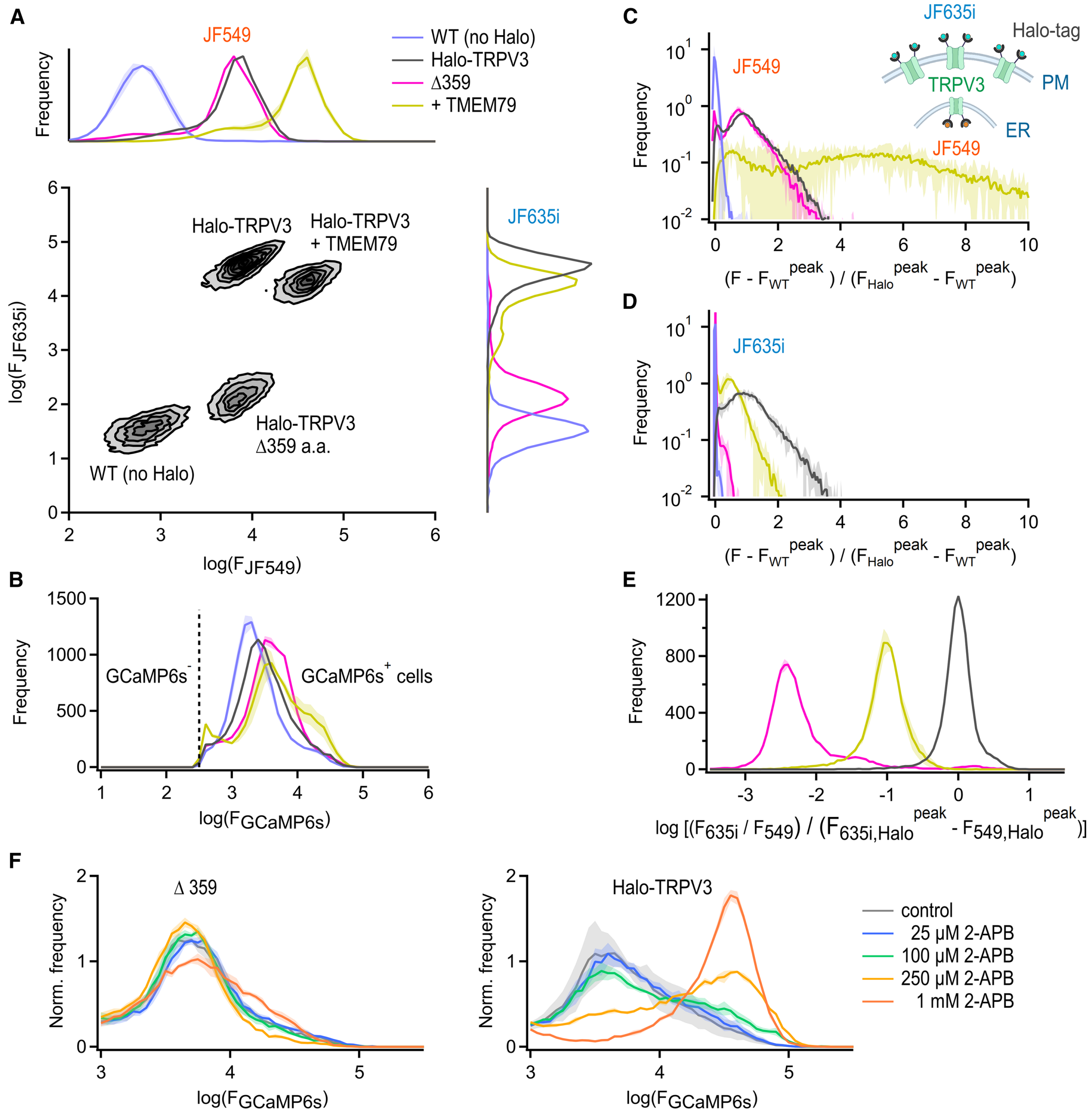

FIGURE 5 Quantitation of surface-expressed vs. intracellular Halo-TRPV3 channels by flow cytometry. (*A*, *top panel*) Histograms showing the logarithmic JF549 fluorescence intensity distributions of single dually labeled GCaMP6s$^+$ cells measured by flow cytometry. Data are shown as mean ± SEM ($n = 3$), with 1×10^4 cells analyzed per replicate. (*A*, *right panel*) Histograms showing the logarithmic JF635i fluorescence intensity distributions measured by flow cytometry of the same cells as in the top panel. Data are shown as mean ± SEM. (*A*, *center panel*) Surface plots depicting the cell distribution as a function of their JF549 and JF635i fluorescence intensities. (*B*) Histograms showing the logarithmic GCaMP6s fluorescence intensity distributions of the same cells as in (*A*) measured by flow cytometry. Data are shown as mean ± SEM. The dashed line indicates the fluorescence cutoff to select GCaMP6s$^+$ cells. (*C*) Histograms of the JF549 fluorescence intensity distribution of cells expressing TRPV3 constructs relative to the peak of the JF549 fluorescence distribution for cells expressing Halo-TRPV3. The peak of the JF549 fluorescence distribution for cells expressing TRPV3 channels without a HaloTag, reflecting nonspecific fluorescence labeling, was subtracted. Data are shown as mean ± 95% confidence interval. (*D*) Histograms of the JF635i fluorescence intensity distribution of cells expressing TRPV3 constructs relative to the peak of the JF635i fluorescence distribution for cells expressing Halo-TRPV3. The peak of the JF635i fluorescence distribution for cells expressing TRPV3 channels without a HaloTag, reflecting nonspecific fluorescence labeling, was subtracted. Data are shown as mean ±95% confidence interval. (*E*) Histograms of the logarithmic subcellular localization ratio between JF635i and JF549 fluorescence intensities in individual cells, normalized by the ratio between the peaks of the JF635i and JF549 fluorescence distribution histograms for cells expressing Halo-TRPV3. Data are shown as mean ± SEM. (*F*) Histograms showing the GCaMP6s fluorescence intensity distribution in the absence or presence of different concentrations of 2-APB, measured in unlabeled cells expressing Halo-TRPV3 Δ359 a.a. (*left*) or Halo-TRPV3 (*right*). Data are shown as mean ± SEM ($n = 3$). Histograms from individual replicates were normalized by the area under the curve after the cutoff at $\log(F_{GCaMP6s}) = 3$.

(Fig. 5 *F*). This indicates that our stable cell line expressing GCaMP6s and Halo-TRPV3 can be effectively used in high-throughput mutational scans to identify variant phenotypes both in terms of their subcellular localization ratio between JF635i and JF549 labeling, and their ion channel activity in response to 2-APB. We also performed one experiment to test whether stable cell lines expressing WT TRPV3 without a HaloTag, Halo-TRPV3, Halo-TRPV3 + TMEM79, and Halo-TRPV3 Δ359 a.a. responded to a fully activating concentration of camphor, another TRPV3 channel agonist. We observed robust responses to the agonist, reflected as an increase in GCaMP6s fluorescence, in all of the cell groups except in those expressing Δ359 a.a. channels (Fig. S7). Furthermore, we observed identical responses in cells expressing WT (no Halo) or Halo-TRPV3 channels, indicating that insertion of the tag does not interfere with channel activation by camphor when assessed using a fluorescent Ca^{2+} reporter.

DISCUSSION

Accurately quantifying subcellular localization of membrane proteins is critical for understanding their "life cycle" and function within cells. The limitations imposed by methods such as surface biotinylation or fluorescent immunolabeling for detecting the dynamics of TRPV3 channel expression in the plasma membrane underscore the critical need for more specific and sensitive tools. To address this gap, we developed a Halo-TRPV3 system that provides distinct advantages over traditional fluorescent proteins or biochemical methods. This includes the ability of HaloTags to bind spectrally separable membrane-permeable and -impermeable fluorescent dyes, which enabled us to label and detect with high specificity channels localized in the cell surface versus in intracellular compartments in live cells using imaging techniques with the capacity to acquire data with high throughput. To verify that the system functions as intended, we 1) confirmed that the HaloTag fusion preserves TRPV3 channel sensitivity to activators; 2) verified the specificity and functionality of the fluorescent dye labeling scheme; 3) validated the sensitivity of this tool to detect partial and severe defects in plasma membrane trafficking caused by co-expression of TRPV3 channels with the ER-resident protein TMEM79 or the deletion of 359 amino acids in the N-terminus of the channel, respectively; 4) co-expressed additional fluorophores to report on TRPV3 activity-dependent Ca^{2+} entry into cells, to label specific subcellular compartments like the plasma membrane of lysosomes, and used the cell viability dye DAPI to identify cells with an intact plasma membrane; and 5) described a rapid prelabeling cell fixation protocol that prevents cell permeabilization, which could be used in combination with our labeling and imaging approach to quantify time-dependent membrane remodeling processes involving TRPV3.

Our first objective was to determine whether the HaloTag fusion into the S5-S6 extracellular domains of TRPV3 interfered with normal channel function. This is particularly important because each TRPV3 channel tetramer includes four HaloTag proteins confined within a relatively small volume directly above the channel pore, raising the possibility that the insertion might completely disrupt channel function. Whole-cell and excised patch-clamp recordings obtained from transiently transfected HEK293 cells as well as iCasp9 stable cell lines revealed that Halo-TRPV3 channels had a similar sensitivity to activation by voltage, 2-APB, and temperature as WT channels without a HaloTag. Furthermore, our experiments show that the HaloTag insertion did not appreciably affect the rapid kinetics of channel activation and deactivation by voltage nor the slow kinetics of current increase that are characteristic of TRPV3 channels when they are exposed to micro-molar concentrations of 2-APB or heat. Interestingly, oversaturating concentrations of 2-APB (1 mM) caused Halo-TRPV3 channels to desensitize more rapidly than WT channels, indicating that the HaloTag insertion causes an alteration in channel desensitization under extreme stimulation conditions. Because of the very high concentration of 2-APB that was required to observe this effect, compared with the largely unaltered responses that we observed under standard experimental conditions (30,60,61), we believe that our HaloTag construct is a suitable tool to study cellular and molecular mechanisms of TRPV3 channel function under standard experimental conditions.

Surface biotinylation is widely used for assessing localization of proteins in the plasma membrane. However, labeling with biotin lacks protein specificity, requires large numbers of cells whose plasma membrane must be in optimal condition, and must be carried out under conditions that minimize membrane remodeling during surface biotinylation. In addition, it involves multiple sample-processing steps that include cell lysis, binding and elution to an affinity matrix, gel electrophoresis, and blot transfer into a membrane for immunodetection, which reduces its overall sensitivity and limits its use for studying dynamic protein trafficking processes. The HaloTag technology circumvents these limitations; its ability to bind affinity beads enables protein-selective labeling and capture for biochemical analysis (41), whereas its labeling with bright and stable membrane-permeable and impermeable dyes allows the detection of labeled proteins directly on SDS-PAGE gels. We show that loading only a small fraction of the total lysate obtained from $<1 \times 10^6$ cells labeled with JF549 (i.e., a single confluent well in a six-well plate) yielded robust in-gel fluorescence bands for Halo-TRPV3 (Fig. S2 *B*). These observations indicate that it should be possible to perform mid-throughput assays where the Halo-TRPV3 channel is used in combination with native or nonreducing gels and cross-linking reagents to detect the formation and stoichiometry of complexes between TRPV3 and other proteins without

requiring immunoblots. Importantly, it is feasible to perform mid-throughput in-gel screens for proteins that interact with TRPV3 using libraries of SNAP-tagged proteins that can be orthogonally labeled with dyes of a different color (67).

To perform quantitative comparisons between cell surface and intracellularly localized TRPV3 channel populations that are not confounded by incomplete labeling with the membrane-impermeable dye JF635i, we established that a concentration of 250 nM of JF635i was sufficient to saturate available extracellular sites using an incubation time of 30 min at 37°C. An additional 30 min at 37°C are required to then label cells with JF549. A caveat of these long incubation periods of live cells with each of the HaloTag dyes is that substantial membrane remodeling could occur at 37°C. Our confocal imaging using both live and fixed cells indicates that membrane remodeling is minimal under our experimental conditions, because the two sets of images do not show any marked differences, at least at the qualitative level. The cell fixation protocol that we describe would make it possible to fix groups of cells at different time points, followed by application of the labeling and imaging approach that we describe here to quantify time-dependent membrane remodeling processes. These types of experiments are critical to determine whether the slow sensitization and desensitization processes that are characteristic of TRPV3 include any contribution from channel internalization or externalization caused by exposure of cells to channel activators.

Our epifluorescence microscopy and flow cytometry data establish that this Halo-TRPV3 channel labeling and analysis approach can be utilized to robustly differentiate between subtle alterations in channel trafficking, such as the partial ER retention caused by TMEM79 (9), and the virtually complete intracellular retention seen in the Δ359 deletion mutant. This underscores the utility of this tool for performing high-throughput mutational screens focusing on TRPV3 channels. These types of unbiased screening approaches are uniquely suited to learn how proteins like TRPV3 operate, in which the sites in the channel that are targeted by most activators remain unclear despite extensive published structural work focusing on these proteins, and where the mechanisms by which channels respond to stimuli involve dynamic allosteric networks that span across the entire protein (3,51,60,68). Importantly, the iCasp9 landing pad system (52) that we used in this study has been widely utilized to express protein variant libraries for high-throughput mutational screens (55–59). Here, it enabled us to stably co-express GCaMP6s and Halo-TRPV3 channels, yielding homogenous and robust calcium responses upon channel stimulation by two different agonists, as well as highly specific and homogenous labeling patterns using JF635i and JF549 dyes across cells expressing HaloTRPV3. Moreover, growing cells in suspension makes it easier to scale up cell culture size to carry out cell sorting experiments of large protein variant libraries, and it contributes to improved cellular health and plasma membrane integrity when resuspending cells before flow cytometry. This system is thus ideal for implementations of Sort-seq (65,66) methods involving flow cytometry and next-generation sequencing to assign phenotypes to TRPV3 channel variants at high throughput based on both channel activity (detected with GCaMP6s), overall protein abundance (calculated as the sum of JF549 and JF635i labeled channels), and cell surface expression based on JF635i labeling. Notably, obtaining readouts for channel activity and cell surface expression in mutational screens is critical for deconvolving how individual amino acid substitutions affect channel gating and trafficking and thus gaining mechanistic insight from the results of the screen. This is of particular relevance for identifying gain-of-function variants because the increased baseline activity in these types of mutants is generally accompanied by a strong reduction in expression (37,60,62,69), making them impossible to identify with just a Ca^{2+}-reporter assay. One important limitation of the iCasp9 landing pad system, however, is that cells expressing gain-of-function variants experience a strong negative selection pressure that results in the loss of cells expressing these variants (not shown). Having the capacity to identify and characterize novel gain-of-function variants of TRPV3 would be impactful because several of these variants have been found to cause Olmsted syndrome in human patients (35,37,70). Additional work is therefore required to identify conditions that favor survival of cells expressing gain-of-function channel variants; promising strategies may include the supplementation of cell cultures with TRPV3-specific inhibitors and maintaining iCasp9 cells in the absence of the tetracycline-promoter inducer that is required for landing pad gene expression.

We have also shown that the landing pad system can be used to express Halo-TRPV3 together with an additional membrane protein, TMEM79, and GCaMP6s, EGFP-PH-PLCδ, or LAMP1-EGFP, without negatively impacting the protein abundance of TRPV3. We observe that co-expression with TMEM79 results in pronounced retention of TRPV3 in intracellular compartments, accompanied by a more modest decrease in cell surface expression of about twofold. Notably, patch-clamp recordings of transiently transfected HEK293 cells expressing TRPV3 with or without TMEM79 show that TMEM79 causes about a twofold reduction in whole-cell current density (9), consistent with our conclusions regarding the effect of this protein on the cell surface expression of TRPV3. One important advantage of the iCasp9 system is that proteins are expressed from the landing pad in fixed proportions that are similar across all cells in the population, whereas the use of transient transfection requires precise balancing between co-transfected plasmids, which can be challenging to consistently replicate. Our confocal imaging using LAMP1-EGFP further support previous observations that TMEM79 increases localization of TRPV3 channels

into lysosomes (9); indeed we observed multiple lysosomes in cells expressing TMEM79 that co-localized with JF549-labeled Halo-TRPV3 channels, whereas we did not observe any in cells without TMEM79. Interestingly, even in cells with TMEM79, we found that a large portion of lysosomes did not co-localize with JF549-labeled Halo-TRPV3, raising the question of what properties define the types of lysosomes that contain Halo-TRPV3 versus those that do not. Together, our findings set the stage for high-throughput mutational screens aimed at dissecting the molecular and cellular mechanisms through which proteins such as TMEM79 influence the activity and trafficking of TRPV3 channels.

It is important to point out that although the work described here was carried out using HEK293-derived cells overexpressing both TRPV3 and TMEM79 proteins, it is possible to transiently transfect Halo-TRPV3 channels into keratinocyte cell lines, neurons, or other cell types of interest, as has been successfully done using transfected sensory neurons to study transport dynamics of voltage-gated Na^{+} channels fused to a HaloTag (44–48). This would permit studies carried out under more physiological conditions to observe how TRPV3 channel expression and transport can be regulated by other cellular and environmental factors. Our observation that the HaloTag insertion does not seem to cause major alterations in channel activity raises the exciting possibility of generating knockin animals expressing this tagged construct in the same types of cells where TRPV3 is physiologically expressed. This would facilitate the identification of cell types other than keratinocytes where TRPV3 channels are also expressed, as well as taking advantage of the diverse toolset of HaloTag ligands to gain an understanding of the roles that this channel plays in living organisms. Thus, this novel protein tool introduces a reliable and versatile reporter to quantitatively assess TRPV3 subcellular localization, providing a powerful approach for dissecting the mechanisms behind TRPV3 function and advancing studies of channel regulation, disease-associated variants, and therapeutic discoveries.

DATA AND CODE AVAILABILITY

All data needed to evaluate the conclusions of this manuscript are present in the article figures or Supporting Material. Raw fluorescence microscopy images, flow cytometry data sets, or gel image scans can be provided upon reasonable request.

ACKNOWLEDGMENTS

We thank Douglas Fowler (University of Washington) and Kenneth Matreyek (Case Western Reserve University) for their gifts of the KAM05_attB_mCherry plasmid and the iCasp9 landing pad cell line. We thank HHMI's Janelia Research Campus for sharing reagents (JF635i). We also thank Richard Salinas at the University of Texas at Austin Center for Biomedical Research Support Microscopy and Flow Cytometry Facility (RRID:SCR_021756) for technical assistance with cell sorting. We thank Husniye Kantarci (UT Austin) for giving us access to her confocal microscope, Dominic Balice, Nikita Elinson-Watson, Karina Peña, and Kimberly Castillo for their assistance with confocal imaging, Helen Chen, Averi Pike, and Rose Hudson for technical assistance and helpful discussions, and Susanne Ressl (UT Austin) and Marcel Goldschen-Ohm (UT Austin) for helpful discussions. We thank Averi Pike, Cailin Mulry, and Renee Stephenson for reading and commenting on the manuscript. This work was supported by UT Austin startup funds to A.J.-O. and E.S.

AUTHOR CONTRIBUTIONS

A.J.-O. conceptualized, supervised, and administered the project. A.J.-O. and E.N.S. acquired funds for the project. A.H., J.C., and A.K. performed molecular cloning. A.H. and J.C. performed tissue culture, fluorescence imaging, and flow cytometry experiments. A.J.-O. performed SDS-PAGE and electrophysiology experiments. A.H., J.C., and A.J.-O. analyzed and curated data and contributed to visualization. A.H. wrote the original draft of the paper. All authors reviewed and edited the paper.

DECLARATION OF INTERESTS

We have no competing interests to declare.

SUPPORTING MATERIAL

Supporting material can be found online at https://doi.org/10.1016/j.bpj.2026.03.003.

REFERENCES

1. Xu, H., I. S. Ramsey, …, D. E. Clapham. 2002. TRPV3 is a calcium-permeable temperature-sensitive cation channel. *Nature.* 418:181–186.
2. Singh, A. K., L. L. McGoldrick, and A. I. Sobolevsky. 2018. Structure and gating mechanism of the transient receptor potential channel TRPV3. *Nat. Struct. Mol. Biol.* 25:805–813.
3. Zubcevic, L., M. A. Herzik, …, S.-Y. Lee. 2018. Conformational ensemble of the human TRPV3 ion channel. *Nat. Commun.* 9:4773.
4. Shimada, H., T. Kusakizako, …, O. Nureki. 2020. The structure of lipid nanodisc-reconstituted TRPV3 reveals the gating mechanism. *Nat. Struct. Mol. Biol.* 27:645–652.
5. Deng, Z., G. Maksaev, …, P. Yuan. 2020. Gating of human TRPV3 in a lipid bilayer. *Nat. Struct. Mol. Biol.* 27:635–644.
6. Peier, A. M., A. J. Reeve, …, A. Patapoutian. 2002. A heat-sensitive TRP channel expressed in keratinocytes. *Science.* 296:2046–2049.
7. Moqrich, A., S. W. Hwang, …, A. Patapoutian. 2005. Impaired thermosensation in mice lacking TRPV3, a heat and camphor sensor in the skin. *Science.* 307:1468–1472.
8. Chung, M.-K., H. Lee, …, M. J. Caterina. 2004. TRPV3 and TRPV4 mediate warmth-evoked currents in primary mouse keratinocytes. *J. Biol. Chem.* 279:21569–21575.
9. Lei, J., R. U. Yoshimoto, …, M. Tominaga. 2023. Involvement of skin TRPV3 in temperature detection regulated by TMEM79 in mice. *Nat. Commun.* 14:4104.
10. Lei, J., and M. Tominaga. 2025. TRPV3 in skin thermosensation and temperature responses. *J. Physiol. Sci.* 75:100005.
11. Huang, S. M., H. Lee, …, M. J. Caterina. 2008. Overexpressed transient receptor potential vanilloid 3 ion channels in skin keratinocytes modulate pain sensitivity via prostaglandin E2. *J. Neurosci.* 28:13727–13737.
12. Szöllősi, A. G., N. Vasas, …, T. Bíró. 2018. Activation of TRPV3 regulates inflammatory actions of human epidermal keratinocytes. *J. Invest. Dermatol.* 138:365–374.

13. Han, Y., A. Luo, …, R. Lai. 2021. A plant-derived TRPV3 inhibitor suppresses pain and itch. *Br. J. Pharmacol.* 178:1669–1683.

14. Qu, Y., S. Mo, …, K. Wang. 2025. Selective inhibition of TRPV3 channel by natural rosmarinic acid and its analogs for alleviation of skin lesions through downregulation of NF-κB pathway. *J. Biol. Chem.* 301:110267.

15. Yamamoto-Kasai, E., K. Imura, …, T. Yoshioka. 2012. TRPV3 as a therapeutic target for itch. *J. Invest. Dermatol.* 132:2109–2112.

16. Zhao, J., A. Munanairi, …, Z.-F. Chen. 2020. PAR2 mediates itch via TRPV3 signaling in keratinocytes. *J. Invest. Dermatol.* 140:1524–1532.

17. Um, J. Y., H. B. Kim, …, H. O. Kim. 2022. TRPV3 and Itch: The Role of TRPV3 in Chronic Pruritus according to Clinical and Experimental Evidence. *Int. J. Mol. Sci.* 23:14962.

18. Cheng, X., J. Jin, …, H. Xu. 2010. TRP channel regulates EGFR signaling in hair morphogenesis and skin barrier formation. *Cell.* 141:331–343.

19. Yamanoi, Y., J. Lei, …, M. Tominaga. 2023. TRPV3-ANO1 interaction positively regulates wound healing in keratinocytes. *Commun. Biol.* 6:88.

20. Aijima, R., B. Wang, …, M. A. Kido. 2015. The thermosensitive TRPV3 channel contributes to rapid wound healing in oral epithelia. *FASEB J.* 29:182–192.

21. Borbíró, I., E. Lisztes, …, T. Bíró. 2011. Activation of transient receptor potential vanilloid-3 inhibits human hair growth. *J. Invest. Dermatol.* 131:1605–1614.

22. Wu, S.-W., J. E. M. Lindberg, and J. H. Peters. 2016. Genetic and pharmacological evidence for low-abundance TRPV3 expression in primary vagal afferent neurons. *Am. J. Physiol. Regul. Integr. Comp. Physiol.* 310:R794–R805.

23. Moussaieff, A., N. Rimmerman, …, R. Mechoulam. 2008. Incensole acetate, an incense component, elicits psychoactivity by activating TRPV3 channels in the brain. *FASEB J.* 22:3024–3034.

24. Hu, J., H. J. Choo, and S.-X. Ma. 2011. Infrared heat treatment reduces food intake and modifies expressions of TRPV3-POMC in the dorsal medulla of obesity prone rats. *Int. J. Hyperthermia.* 27:708–716.

25. McGaraughty, S., K. L. Chu, …, J.-D. Brederson. 2017. TRPV3 modulates nociceptive signaling through peripheral and supraspinal sites in rats. *J. Neurophysiol.* 118:904–916.

26. Liu, Y., H. Qi, …, H. Sun. 2018. Transient receptor potential vanilloid-3 (TRPV3) activation plays a central role in cardiac fibrosis induced by pressure overload in rats via TGF-β1 pathway. *Naunyn-Schmiedebergs Arch Pharmacol.* 391:131–143.

27. Zhang, Q., H. Qi, …, H. Sun. 2018. Activation of transient receptor potential vanilloid 3 channel (TRPV3) aggravated pathological cardiac hypertrophy via calcineurin/NFATc3 pathway in rats. *J. Cell Mol. Med.* 22:6055–6067.

28. Xu, H., M. Delling, …, D. E. Clapham. 2006. Oregano, thyme and clove-derived flavors and skin sensitizers activate specific TRP channels. *Nat. Neurosci.* 9:628–635.

29. Vogt-Eisele, A. K., K. Weber, …, H. Hatt. 2007. Monoterpenoid agonists of TRPV3. *Br. J. Pharmacol.* 151:530–540.

30. Gochman, A., X.-F. Tan, …, A. Jara-Oseguera. 2023. Cannabidiol sensitizes TRPV2 channels to activation by 2-APB. *eLife.* 12:e86166.

31. Chung, M.-K., H. Lee, …, M. J. Caterina. 2004. 2-aminoethoxydiphenyl borate activates and sensitizes the heat-gated ion channel TRPV3. *J. Neurosci.* 24:5177–5182.

32. Hu, H.-Z., Q. Gu, …, M. X. Zhu. 2004. 2-aminoethoxydiphenyl borate is a common activator of TRPV1, TRPV2, and TRPV3. *J. Biol. Chem.* 279:35741–35748.

33. Li, C., W. Ma, …, Z. Li. 2016. Sorting nexin 11 regulates lysosomal degradation of plasma membrane TRPV3. *Traffic.* 17:500–514.

34. Huang, H.-L., C. Li, …, Z. Li. 2020. Sorting nexin 11 knockout mice exhibit enhanced thermosensing behaviour. *Genes Brain Behav.* 19:e12625.

35. Lin, Z., Q. Chen, …, Y. Yang. 2012. Exome sequencing reveals mutations in TRPV3 as a cause of Olmsted syndrome. *Am. J. Hum. Genet.* 90:558–564.

36. Eytan, O., D. Fuchs-Telem, …, E. Sprecher. 2014. Olmsted syndrome caused by a homozygous recessive mutation in TRPV3. *J. Invest. Dermatol.* 134:1752–1754.

37. Zhong, W., L. Hu, …, Y. Yang. 2021. Genotype−Phenotype Correlation of TRPV3-Related Olmsted Syndrome. *J. Invest. Dermatol.* 141:545–554.

38. Los, G. V., L. P. Encell, …, K. V. Wood. 2008. HaloTag: a novel protein labeling technology for cell imaging and protein analysis. *ACS Chem. Biol.* 3:373–382.

39. Encell, L. P., R. Friedman Ohana, …, K. V. Wood. 2012. Development of a dehalogenase-based protein fusion tag capable of rapid, selective and covalent attachment to customizable ligands. *Curr. Chem. Genomics.* 6:55–71.

40. Urh, M., and M. Rosenberg. 2012. Halotag, a platform technology for protein analysis. *Curr. Chem. Genomics.* 6:72–78.

41. England, C. G., H. Luo, and W. Cai. 2015. HaloTag technology: a versatile platform for biomedical applications. *Bioconjug. Chem.* 26:975–986.

42. Cook, A., F. Walterspiel, and C. Deo. 2023. HaloTag-Based Reporters for Fluorescence Imaging and Biosensing. *Chembiochem.* 24:e202300022.

43. Kompa, J., J. Bruins, …, K. Johnsson. 2023. Exchangeable HaloTag Ligands for Super-Resolution Fluorescence Microscopy. *J. Am. Chem. Soc.* 145:3075–3083.

44. Akin, E. J., G. P. Higerd-Rusli, …, S. D. Dib-Hajj. 2019. Building sensory axons: Delivery and distribution of NaV1.7 channels and effects of inflammatory mediators. *Sci. Adv.* 5:eaax4755.

45. Akin, E. J., M. Alsaloum, …, S. D. Dib-Hajj. 2021. Paclitaxel increases axonal localization and vesicular trafficking of Nav1.7. *Brain.* 144:1727–1737.

46. Higerd-Rusli, G. P., S. Tyagi, …, S. D. Dib-Hajj. 2023. The fates of internalized NaV1.7 channels in sensory neurons: Retrograde cotransport with other ion channels, axon-specific recycling, and degradation. *J. Biol. Chem.* 299:102816.

47. Tyagi, S., N. Sarveswaran, …, S. D. Dib-Hajj. 2023. Conserved but not critical: Trafficking and function of NaV1.7 are independent of highly conserved polybasic motifs. *Front. Mol. Neurosci.* 16:1161028.

48. Tyagi, S., G. P. Higerd-Rusli, …, S. D. Dib-Hajj. 2024. Compartment-specific regulation of NaV1.7 in sensory neurons after acute exposure to TNF-α. *Cell Rep.* 43:113685.

49. Deo, C., A. S. Abdelfattah, …, E. R. Schreiter. 2021. The HaloTag as a general scaffold for far-red tunable chemigenetic indicators. *Nat. Chem. Biol.* 17:718–723.

50. Mott, T. M., G. C. Wulffraat, …, E. N. Senning. 2024. Fluorescence labeling strategies for cell surface expression of TRPV1. *J. Gen. Physiol.* 156:e202313523.

51. Nadezhdin, K. D., A. Neuberger, …, A. I. Sobolevsky. 2021. Structural mechanism of heat-induced opening of a temperature-sensitive TRP channel. *Nat. Struct. Mol. Biol.* 28:564–572.

52. Matreyek, K. A., J. J. Stephany, …, D. M. Fowler. 2020. An improved platform for functional assessment of large protein libraries in mammalian cells. *Nucleic Acids Res.* 48:e1.

53. Chen, T.-W., T. J. Wardill, …, D. S. Kim. 2013. Ultrasensitive fluorescent proteins for imaging neuronal activity. *Nature.* 499:295–300.

54. Schneider, C. A., W. S. Rasband, and K. W. Eliceiri. 2012. NIH Image to ImageJ: 25 years of image analysis. *Nat. Methods.* 9:671–675.

55. Howard, M. K., N. Hoppe, …, A. Manglik. 2025. Molecular basis of proton sensing by G protein-coupled receptors. *Cell.* 188:671–687.e20.

56. Coyote-Maestas, W., D. Nedrud, …, D. Schmidt. 2022. Determinants of trafficking, conduction, and disease within a K+ channel revealed through multiparametric deep mutational scanning. *eLife.* 11:e76903.

57. O'Neill, M. J., C.-A. Ng, …, B. M. Kroncke. 2024. Multiplexed Assays of Variant Effect and Automated Patch Clamping Improve

KCNH2-LQTS Variant Classification and Cardiac Event Risk Stratification. *Circulation.* 150:1869–1881.

58. Popp, N. A., R. L. Powell, …, D. M. Fowler. 2025. Multiplex and multimodal mapping of variant effects in secreted proteins via MultiSTEP. *Nat. Struct. Mol. Biol.* 32:2099.
59. Kamath, N. D., and K. A. Matreyek. 2025. Comprehensive mutational characterization of the calcium-sensing STIM1 EF-hand reveals residues essential for structure and function. *Genetics.* 231:iyaf146.
60. Zubcevic, L., W. F. Borschel, …, S.-Y. Lee. 2019. Regulatory switch at the cytoplasmic interface controls TRPV channel gating. *eLife.* 8:e47746.
61. Liu, B., J. Yao, …, F. Qin. 2011. Hysteresis of gating underlines sensitization of TRPV3 channels. *J. Gen. Physiol.* 138:509–520.
62. Yadav, M., and C. Goswami. 2017. TRPV3 mutants causing Olmsted Syndrome induce impaired cell adhesion and nonfunctional lysosomes. *Channels.* 11:196–208.
63. Wulffraat, G. C., S. Mamathasateesh, …, E. N. Senning. 2025. Missense variant analysis in the TRPV1 ARD reveals the unexpected functional significance of a methionine. *PLoS One.* 20:e0331224.
64. Doñate-Macian, P., M. Bañó-Polo, …, A. Perálvarez-Marín. 2015. Molecular and topological membrane folding determinants of transient receptor potential vanilloid 2 channel. *Biochem. Biophys. Res. Commun.* 462:221–226.
65. Araya, C. L., and D. M. Fowler. 2011. Deep mutational scanning: assessing protein function on a massive scale. *Trends Biotechnol.* 29:435–442.
66. Peterman, N., and E. Levine. 2016. Sort-seq under the hood: implications of design choices on large-scale characterization of sequence-function relations. *BMC Genom.* 17:206.
67. Jatzlau, J., W. Burdzinski, …, P. Knaus. 2023. A versatile Halo- and SNAP-tagged BMP/TGFβ receptor library for quantification of cell surface ligand binding. *Commun. Biol.* 6:34.
68. Zhang, F., K. J. Swartz, and A. Jara-Oseguera. 2019. Conserved allosteric pathways for activation of TRPV3 revealed through engineering vanilloid-sensitivity. *eLife.* 8:e42756.
69. Duo, L., L. Hu, …, Y. Yang. 2018. TRPV1 gain-of-function mutation impairs pain and itch sensations in mice. *Mol. Pain.* 14:1744806918762031.
70. Mo, R., X. Ma, …, Z. Chen. 2024. The cumulative effect of compound heterozygous variants in TRPV3 caused Olmsted syndrome. *J. Dermatol. Sci.* 116:111–119.

Biophysical Journal, Volume *125*

Supplemental information

HaloTag-based approach to quantify subcellular localization of TRPV3 channels

Alexander Holloway, Joshua Chiang, Afroza Khan, Eric N. Senning, and Andrés Jara-Oseguera

SUPPORTING MATERIAL

HaloTag-based approach to quantify subcellular localization of TRPV3 channels.

Alexander Holloway, Joshua Chiang, Afroza Khan, Eric N. Senning, and Andrés Jara-Oseguera.

<u>Contents</u>:

Figure S1

Figure S2

Figure S3

Figure S4

Figure S5

Figure S6

Figure S7

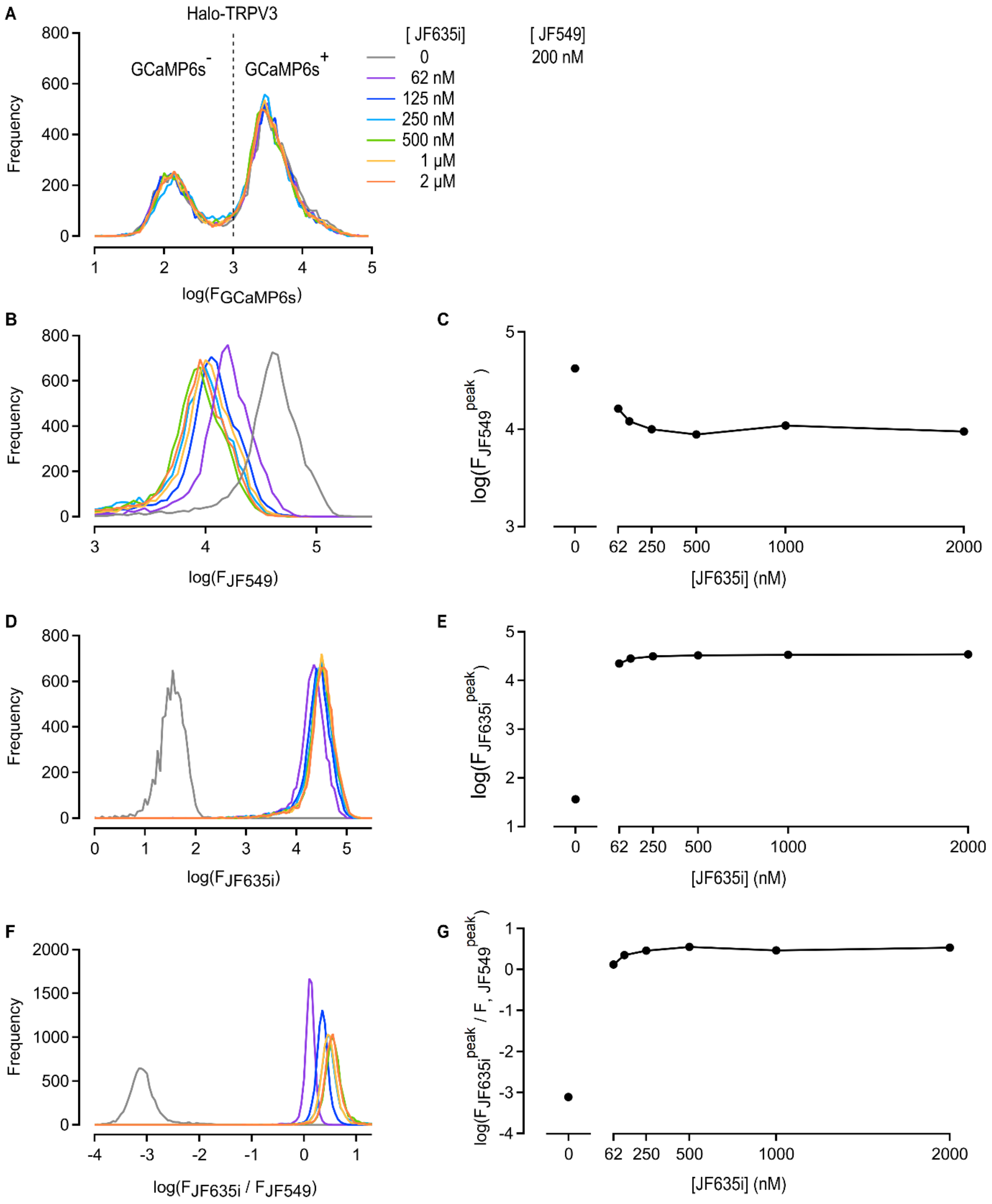

Figure S1. Labeling titration of surface-exposed Halo-TRPV3 channels with JF635i. Quantitative comparison of seven populations of cells expressing Halo-TRPV3 channels titrated

with JF635i at a concentration of 0 nM, 62 nM, 125 nM, 250 nM, 500 nM, 1 μM, or 2 μM before subsequent labeling with JF549 at a concentration of 200 nM. Cells were analyzed by flow cytometry and the Gaussian means of the corresponding histograms were plotted on a saturation curve. **(A, B, D)** Histograms showing the fluorescence intensity distributions for GCaMP6s (A), JF549 (B), and JF635i (D) of cells expressing Halo-TRPV3, measured by flow cytometry. Cells were labeled with 200 nM JF549 and different concentrations of JF635i, as indicated by the trace colors. A total of 1×10^4 cells were analyzed per experimental condition. Histograms in A contain data for all cells, and histograms in (B) and (D) display only data for GCaMP6s$^+$ cells. The dashed line in (A) indicates the GCaMP6s fluorescence intensity cutoff to select for GCaMP6s$^+$ cells in (B) and (D). **(C, E)** JF635i label titration curves of JF549 (C) and JF635i (E) peak fluorescence intensity as a function of the concentration of JF635i used for labeling, obtained from fits of a Gaussian function to the histograms in (B) or (D). **(F)** Histograms of the logarithmic ratio between JF549 and JF635i fluorescence intensities, calculated from the data in (B) and (D). **(G)** JF635i label titration curves of the peak logarithmic ratio between the fluorescence intensities of JF549 and JF635i as a function of the JF635i dye concentration, obtained from fits of a Gaussian function to the histograms in (F).

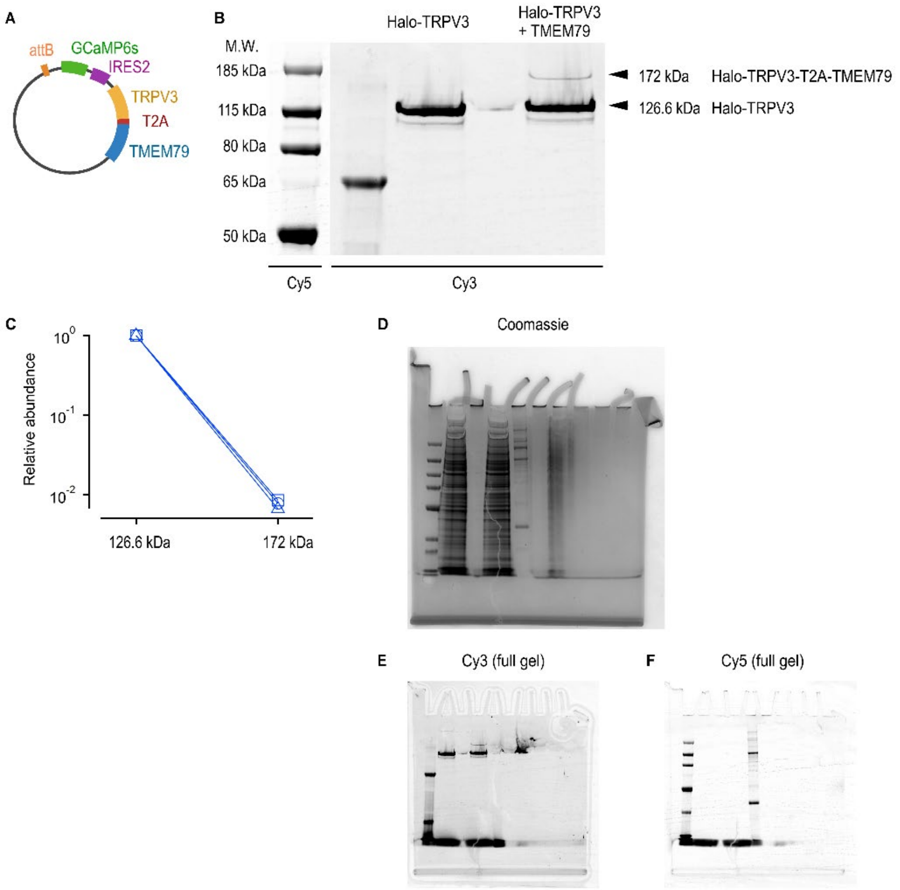

Figure S2. Validation of the use of a T2A sequence to co-express Halo-TRPV3 and TMEM79 in landing pad cells. (A) Cartoon of the promoter-less plasmid that we used to express GCaMP6s, Halo-TRPV3, and TMEM79 in iCasp9 landing pad cells (52). A T2A sequence (dark red) was introduced between the TRPV3 and TMEM79 coding sequences. **(B)** Representative in-gel fluorescence of the total cell lysate obtained from cells expressing Halo-TRPV3 or Halo-TRPV3-T2A-TMEM79 and that were labeled with JF549. The arrows denote bands with molecular weights (M.W.) corresponding to a single Halo-TRPV3 subunit or a Halo-TRPV3-T2A-TMEM79 fusion protein. The molecular weight marker on the same gel was imaged in the Cy5 channel (left panel) and the Cy3 channel (first lane on the right panel). **(C)** Relative abundance of the single

Halo-TRPV3 protein and the Halo-TRPV3-T2A-TMEM79 fusion protein relative to the total labeled Halo-TRPV3 in the gel for lysates of cells recombined with the plasmid for TMEM79 co-expression. Data for three replicate experiments are shown. **(D)** Coomassie staining of the same gel as in (B). **(E)** In-gel fluorescence for the whole gel shown in (B) imaged on the Cy3 channel that shows JF549 fluorescence. **(F)** In-gel fluorescence for the whole gel shown in (B) imaged on the Cy5 channel that shows most bands of the M.W. marker.

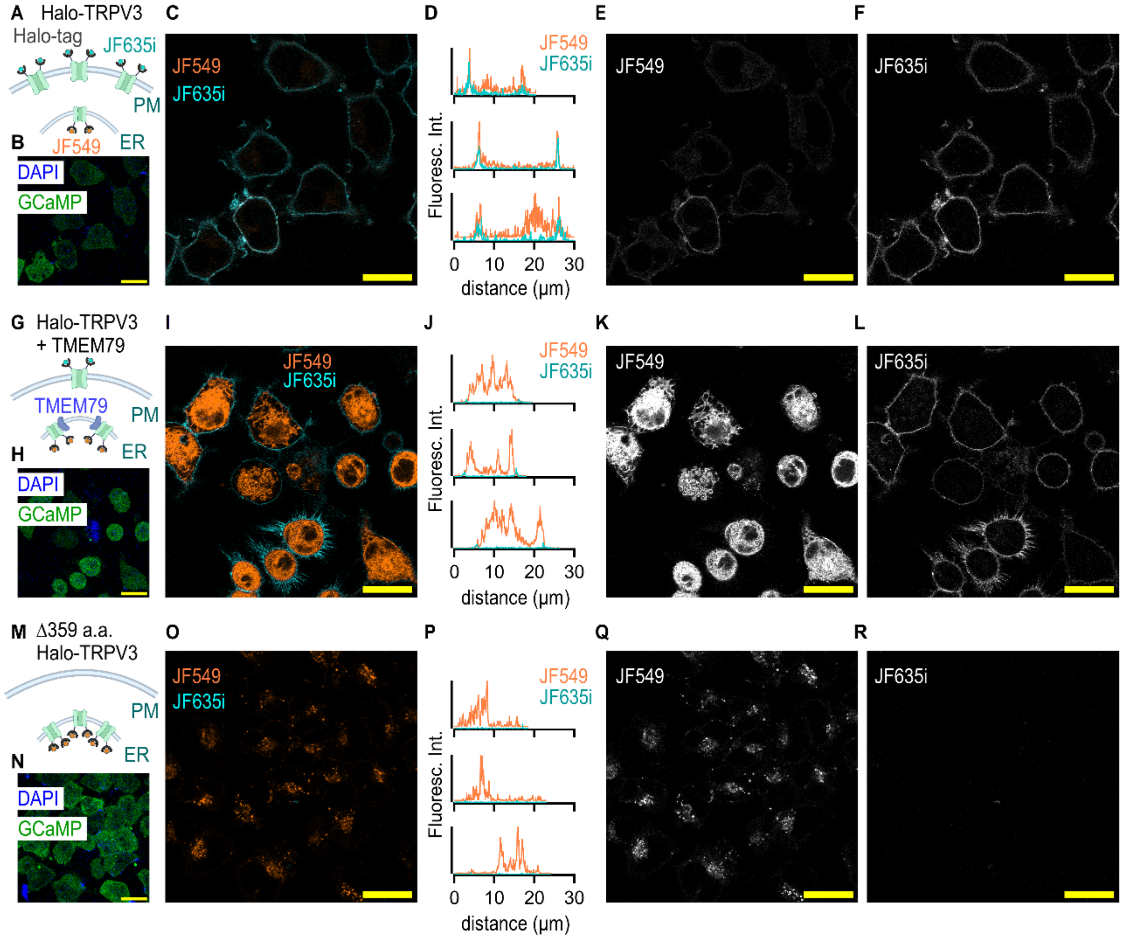

Figure S3. Visualization of Halo-TRPV3 subcellular localization by confocal microscopy in fixed cells. (A, G, and M) Cartoons depicting subcellular localization of Halo-TRPV3 (A), Halo-TRPV3 + TMEM79 (G), and Halo-TRPV3 Δ359 a.a. (M) as determined by our labeling approach applied to fixed cells, using a membrane impermeable dye (cyan, JF635i) followed by a membrane-permeable dye (orange, JF549). PM stands for plasma membrane and ER for endoplasmic reticulum and other intracellular compartments. **(B, H, and N)** Representative GCaMP6s (green) and DAPI (blue) overlaid confocal microscopy images of cells that were fixed before labeling with JF635i, JF549, and DAPI. Images were acquired using a 60x objective. Scale

bars = 20 μm. **(C, I, and O)** Representative JF549 (orange) and JF635i (cyan) overlaid confocal microscopy images of the same fields of view as in (B, H, and N). **(D, J, and P)** JF635i (cyan) and JF549 (orange) fluorescence intensity line profiles from three representative confocal microscopy images of fixed cells expressing Halo-TRPV3 (D), Halo-TRPV3 + TMEM79 (J), or Halo-TRPV3 Δ359 a.a. (P). **(E, K, and Q)** Representative JF549 confocal microscopy images of the same fields of view as in (B, H, and N). **(F, L, and R)** Representative JF635i confocal microscopy images of the same fields of view as in (B, H, and N).

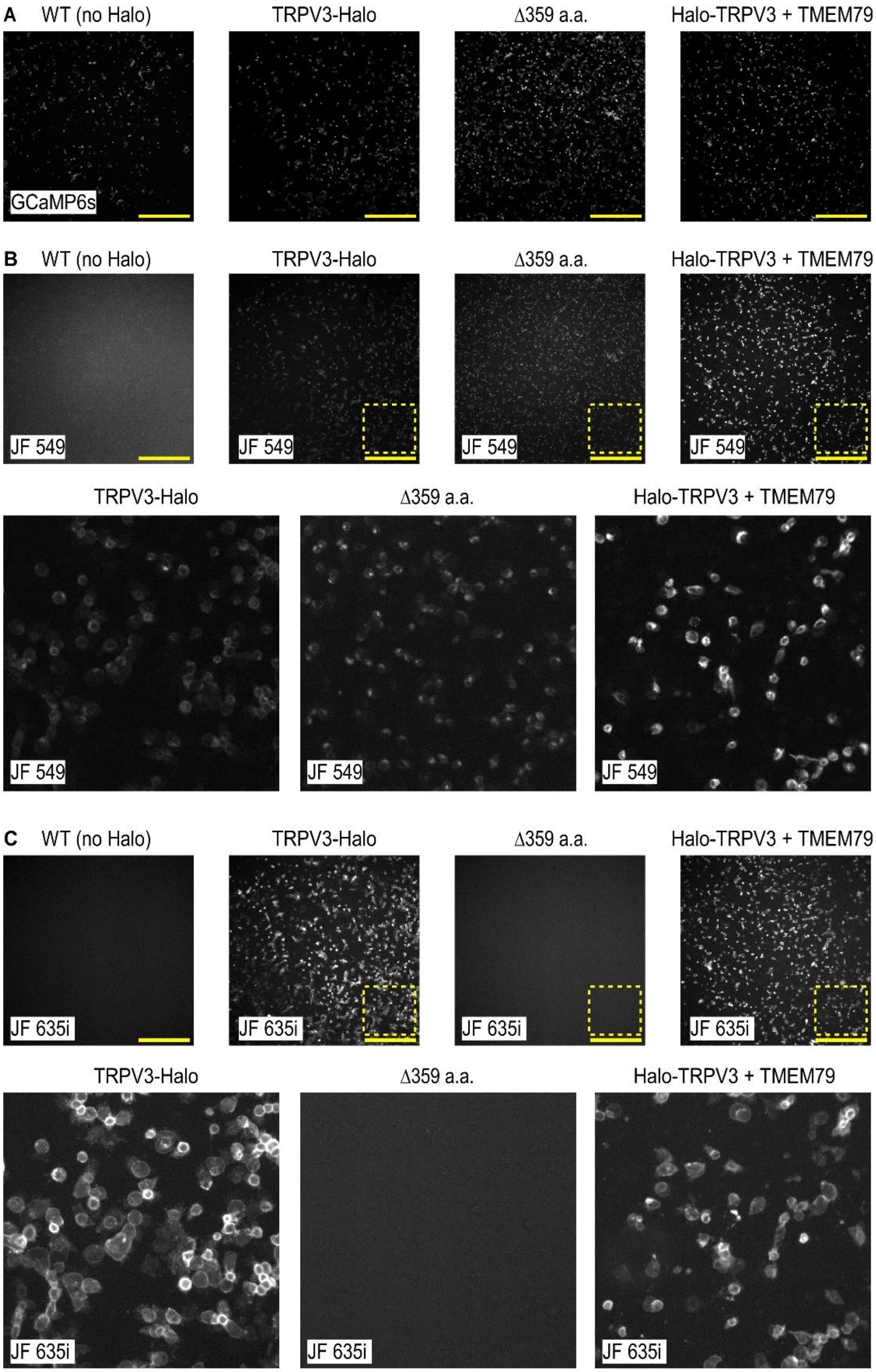

Figure S4. Visualization of Halo-TRPV3 subcellular localization by epifluorescence microscopy in live cells. (A) Representative GCaMP6s fluorescence images obtained by

epifluorescence microscopy of live cells labeled with JF635i and JF549. Images were acquired using a 10x objective. Scale bar is 400 μm. **(B)** Representative JF549 fluorescence images obtained by epifluorescence microscopy of live cells labeled with JF635i and JF549. Images were acquired using a 10x objective. The bottom panel shows magnified images of the regions denoted by the dashed yellow boxes. **(C)** Representative JF635i fluorescence images obtained by epifluorescence microscopy of live cells labeled with JF635i and JF549. Images were acquired using a 10x objective. The bottom panel shows magnified images of the regions denoted by the dashed yellow boxes.

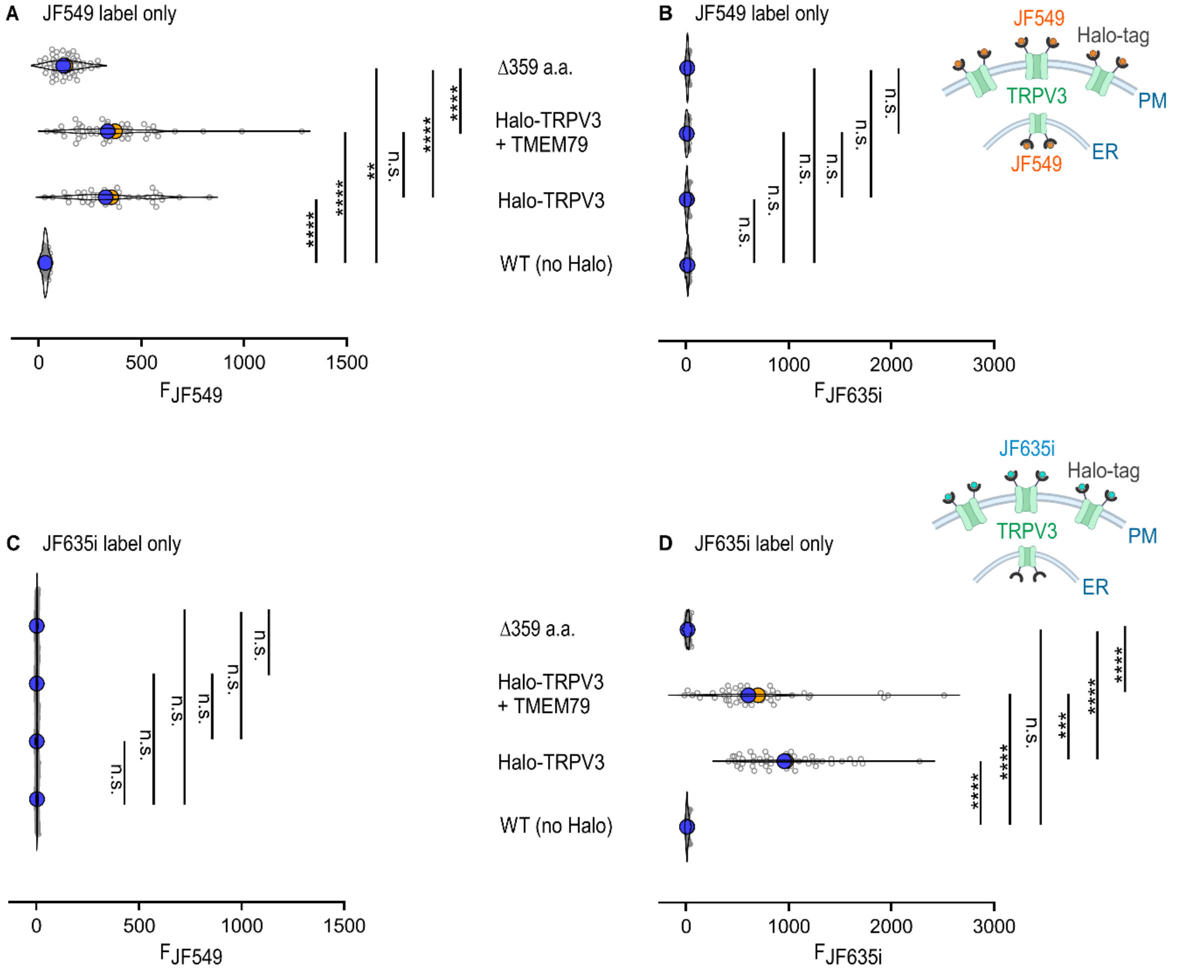

Figure S5. Quantitation of surface-expressed vs intracellular Halo-TRPV3 channels by epifluorescence microscopy in cells labeled with just one of the two dyes. (A) Violin plots of the mean JF549 fluorescence intensity of individual cells measured from epifluorescence microscopy images of cells labeled with JF549 only. Plots contain data from a single experiment, where fifty cells were randomly chosen based on the presence of GCaMP6s fluorescence. Data for individual cells are shown as open gray circles, and the mean and median are shown as yellow and blue circles, respectively. Statistical significance was evaluated using a Tukey HSD test (Halo vs WT, $p = 2.20 \times 10^{-13}$; Halo vs Δ359, $p = 3.84 \times 10^{-10}$; Halo vs TMEM79, $p = 0.93$; TMEM79 vs WT, $p = 1.77 \times 10^{-14}$; TMEM79 vs Δ359, $p = 2.71 \times 10^{-11}$; WT vs Δ359, $p = 0.0054$). **(B)** Violin

plots of the mean JF635i fluorescence intensity of the same cells as in (A). Data are depicted as in (A). Statistical significance was evaluated using a Tukey HSD test (Halo vs WT, $p = 0.917$; Halo vs Δ359, $p = 0.68$; Halo vs TMEM79, $p = 0.645$; TMEM79 vs WT, $p = 0.22$; TMEM79 vs Δ359, $p = 0.075$; WT vs Δ359, $p = 0.959$). **(C)** Violin plots of the mean JF549 fluorescence intensity of individual cells measured from epifluorescence microscopy images of cells labeled with JF635i only. Plots contain data from a single experiment, where fifty cells were randomly chosen based on the presence of GCaMP6s fluorescence. Data are depicted as in (A). Statistical significance was evaluated using a Tukey HSD test (Halo vs WT, $p = 0.533$; Halo vs Δ359, $p = 0.977$; Halo vs TMEM79, $p = 0.752$; TMEM79 vs WT, $p = 0.092$; TMEM79 vs Δ359, $p = 0.934$; WT vs Δ359, $p = 0.299$). **(B)** Violin plots of the mean JF636i fluorescence intensity of the same cells as in (C). Data are depicted as in (A). Statistical significance was evaluated using a Tukey HSD test (Halo vs WT, $p = 7.57 \times 10^{-14}$; Halo vs Δ359, $p = 7.57 \times 10^{-14}$; Halo vs TMEM79, $p = 0.000169$; TMEM79 vs WT, $p = 1.72 \times 10^{-13}$; TMEM79 vs Δ359, $p = 1.86 \times 10^{-13}$; WT vs Δ359, $p = 1$).

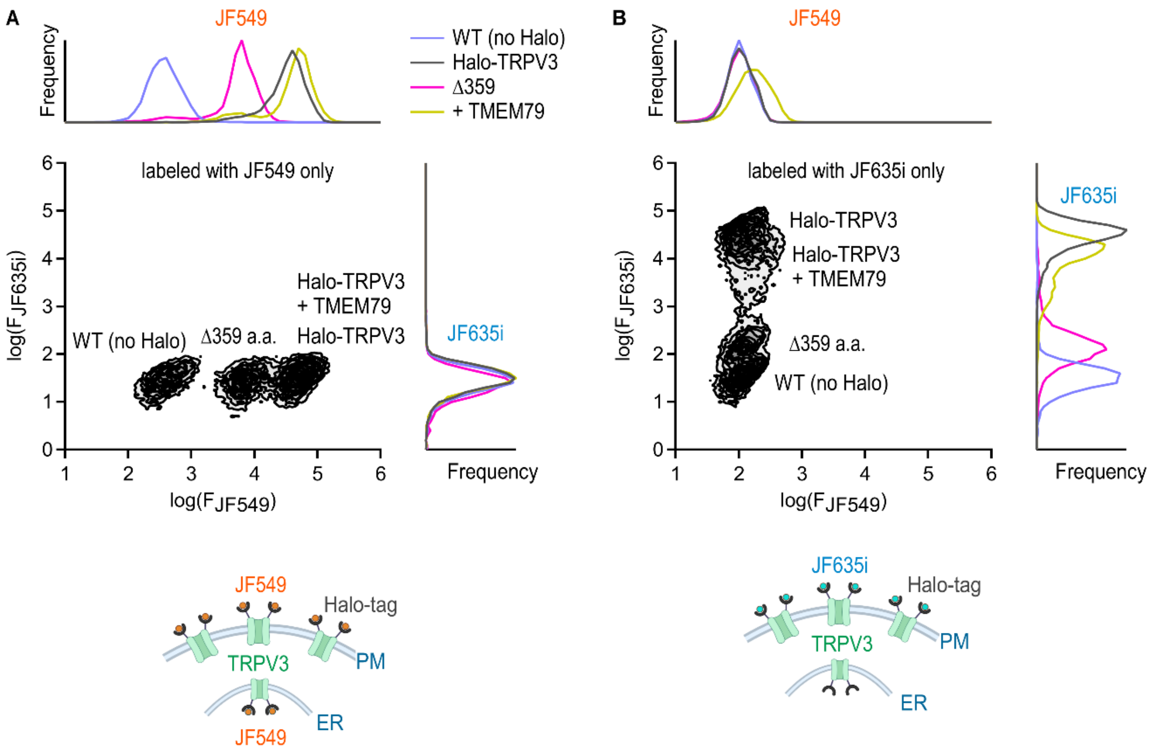

Figure S6. Quantitation of surface-expressed vs intracellular Halo-TRPV3 channels by flow cytometry in cells labeled with just one of the two dyes. **(A**, top panel**)** Histograms showing the logarithmic JF549 fluorescence intensity distributions of single GCaMP6s$^+$ cells labeled with JF549 only and measured by flow cytometry. Data for a single experiment is shown, with 1×10^4 analyzed GCaMP6s$^+$ single cells. **(A**, right panel**)** Histograms showing the logarithmic JF635i fluorescence intensity distributions measured by flow cytometry of the same cells as in the top panel. **(A**, center panel**)** Surface plots depicting the cell distribution as a function of their JF549 and JF635i fluorescence intensities. **(B**, top panel**)** Histograms showing the logarithmic JF549 fluorescence intensity distributions of single GCaMP6s$^+$ cells labeled with JF635i only and measured by flow cytometry. Data for a single experiment is shown, with 1×10^4 analyzed GCaMP6s$^+$ single cells. **(B**, right panel**)** Histograms showing the logarithmic JF635i fluorescence intensity distributions measured by flow cytometry of the same cells as in the top panel. **(B**, center

panel**)** Surface plots depicting the cell distribution as a function of their JF549 and JF635i fluorescence intensities.

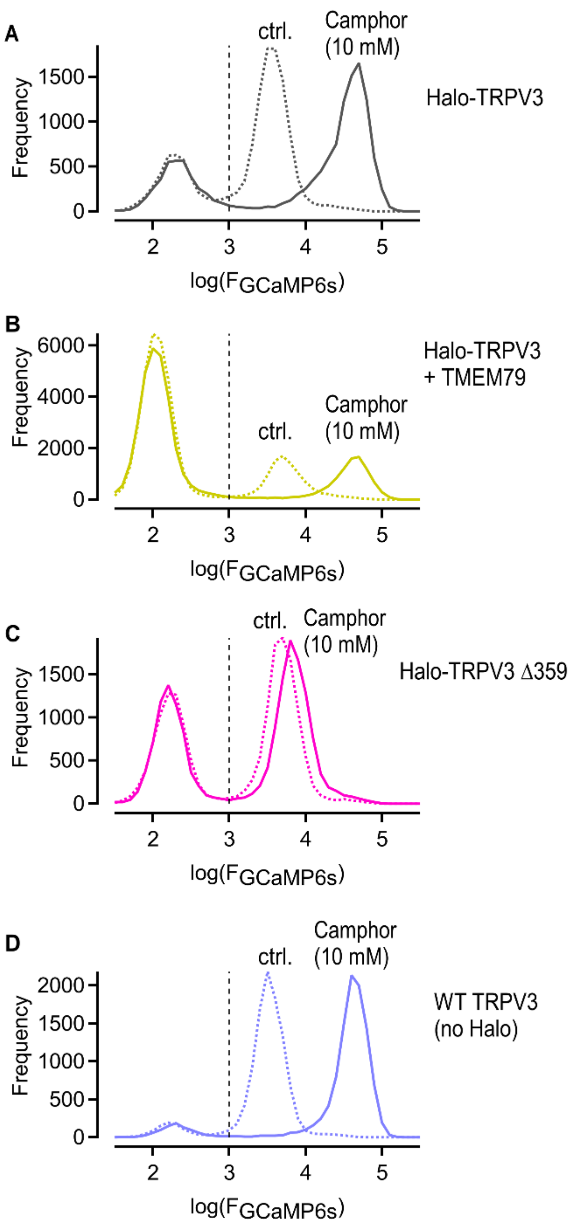

Figure S7. Measuring the response of TRPV3 channels to camphor with a fluorescent calcium reporter. (A-D) Histograms showing the GCaMP6s fluorescence intensity distribution in the presence (solid curves) or absence (dotted curves) of 10 mM camphor of unlabeled cells expressing Halo-TRPV3 (A, gray), Halo-TRPV3 + TMEM79 (B, lime green), Halo-TRPV3 Δ359 a.a. (C, pink), and WT TRPV3 (no Halo) (D, light blue). A total of 1×10^4 cells were analyzed per

experimental group. The dashed vertical line distinguishes between GCaMP6s^{-} and GCaMP6s^{+} cell populations.
